# Quantification of Microbial Source Tracking and Pathogenic Bacterial Markers in Water and Sediments of Tiaoxi River (Taihu Watershed)

**DOI:** 10.3389/fmicb.2019.00699

**Published:** 2019-04-24

**Authors:** Kiran Kumar Vadde, Alan J. McCarthy, Rong Rong, Raju Sekar

**Affiliations:** ^1^Department of Biological Sciences, Xi’an Jiaotong-Liverpool University, Suzhou, China; ^2^Microbiology Research Group, Institute of Integrative Biology, University of Liverpool, Liverpool, United Kingdom

**Keywords:** fecal pollution, Taihu watershed, Tiaoxi River, microbial source tracking (MST), pathogens, qPCR quantification

## Abstract

Taihu Lake is one of the largest freshwater lakes in China, serving as an important source of drinking water; >60% of source water to this lake is provided by the Tiaoxi River. This river faces serious fecal contamination issues, and therefore, a comprehensive investigation to identify the sources of fecal contamination was carried out and is presented here. The performance of existing universal (BacUni and GenBac), human (HF183-Taqman, HF183-SYBR, BacHum, and Hum2), swine (Pig-2-Bac), ruminant (BacCow), and avian (AV4143 and GFD) associated microbial source tracking (MST) markers was evaluated prior to their application in this region. The specificity and sensitivity results indicated that BacUni, HF183-TaqMan, Pig-2-Bac, and GFD assays are the most suitable in identifying human and animal fecal contamination. Therefore, these markers along with marker genes specific to selected bacterial pathogens were quantified in water and sediment samples of the Tiaoxi River, collected from 15 locations over three seasons during 2014 and 2015. Total/universal *Bacteroidales* markers were detected in all water and sediment samples (mean concentration 6.22 log_10_ gene copies/100 ml and 6.11 log_10_ gene copies/gram, respectively), however, the detection of host-associated MST markers varied. Human and avian markers were the most frequently detected in water samples (97 and 89%, respectively), whereas in sediment samples, only human-associated markers were detected more often (86%) than swine (64%) and avian (8.8%) markers. The results indicate that several locations in the Tiaoxi River are heavily polluted by fecal contamination and this correlated well with land use patterns. Among the five bacterial pathogens tested, *Shigella* spp. and *Campylobacter jejuni* were the most frequently detected pathogens in water (60% and 62%, respectively) and sediment samples (91% and 53%, respectively). Shiga toxin-producing *Escherichia coli* (STEC) and pathogenic *Leptospira* spp. were less frequently detected in water samples (55% and 33%, respectively) and sediment samples (51% and 13%, respectively), whereas *E. coli* O157:H7 was only detected in sediment samples (11%). Overall, the higher prevalence and concentrations of *Campylobacter jejuni, Shigella* spp., and STEC, along with the MST marker detection at a number of locations in the Tiaoxi River, indicates poor water quality and a significant human health risk associated with this watercourse.

**GRAPHICAL ABSTRACT:**
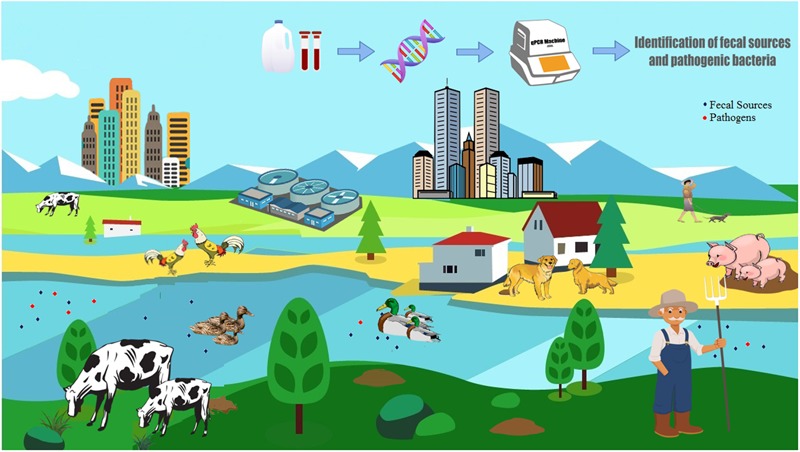
Tracking fecal contamination and pathogens in watersheds using molecular methods.

Tracking fecal contamination and pathogens in watersheds using molecular methods.

## Introduction

Fecal contamination of drinking water sources, harvestable shellfish, and recreational waters is a major concern of public health, as it promotes human exposure to pathogenic microorganisms (Napier et al., 2017). Therefore, continuous monitoring and proper protection of these waters are required. Traditionally, fecal indicator bacteria (FIB) have been used to monitor pollution in environmental waters and to assess the associated public health risks (Griffith et al., 2009). There are several limitations to using FIB for microbial water quality monitoring, as these bacteria can persist and replicate outside of the host (Byappanahalli et al., 2003) and there can also be a poor correlation between FIB and pathogen presence (Ahmed et al., 2013). The major limiting factor, however, is that FIB detection does not indicate the source or origin of fecal contamination (Field and Samadpour, 2007), which is crucial for the characterization of a public health risk and the implementation of remediation measures. Therefore, microbial source tracking (MST) techniques have been developed over the last decade to unequivocally identify the sources and origins of fecal pollution. Both library-dependent (LD-MST) and library-independent MST (LI-MST) methods are available for identifying the source of fecal pollution, although LD-MST methods have several limitations in correctly assigning fecal contamination to host-specific sources (Harwood et al., 2003; Field and Samadpour, 2007). However, quantitative PCR (qPCR) based LI-MST techniques have proven to be widely applicable in the study of fecal contamination in environmental samples, because they can accurately quantify the host-specific MST target sequences (Layton et al., 2006; Kildare et al., 2007). Among the LI-MST methods, *Bacteroidales* are often used as the target, as they are obligate anaerobic bacteria found in the human and animal gut at higher levels than *E. coli* (Bernhard and Field, 2000); host-associated *Bacteroidales* 16S rRNA gene markers have been developed for different hosts to discriminate between human and other animal fecal sources in the environment (Kildare et al., 2007; Shanks et al., 2008; Mieszkin et al., 2009; Raith et al., 2013; Green et al., 2014). It has recently been reported that avian feces could be better distinguished from other fecal sources by targeting bacterial taxonomic groups such as *Helicobacter* spp., rather than *Bacteroidales* 16S rRNA (Green et al., 2012).

Previous reports specified that geographical differences could significantly affect the performance of these MST assays and recommended proper assessment prior to field application at new study areas (Reischer et al., 2013; Odagiri et al., 2015; Boehm et al., 2016). In China, studies on qPCR based MST assays to monitor fecal pollution are very limited (Jia et al., 2014; He et al., 2016; Fan et al., 2017). He et al. (2016) validated five MST and four mitochondrial DNA fecal source tracking (FST) markers for their applicability to study fecal pollution in the Taihu Lake watershed. They reported that mitochondrial DNA based human FST methods were superior (though slight cross-reactivity was observed with pig fecal samples, the primary livestock in the Taihu watershed) to those *Bacteroidales* based MST (BacH, HF183 SYBR) assays tested, but the most widely used MST makers, such as HF183 Taqman and BacHum qPCR assays were not included. They also reported that the Pig-2-Bac assay (*Bacteroidales* based) showed a higher accuracy than other mitochondrial DNA based swine FST markers. Jia et al. (2014) used swine specific *Bacteroidales* assay (Pig-2-Bac) to assess the levels of swine fecal pollution in the Hongqi River, and Fan et al. (2017) developed two new assays based on genome fragment enrichment (GFE) targeting *Bacteroidales*-like sequences present in pig fecal samples. Although MST methods provide information on the source of fecal pollution, they do not provide evidence for, or confirm the presence of, bacterial pathogens. Determining the correlation between MST data and direct pathogen detection has been addressed in relatively few cases (Ridley et al., 2014; Bradshaw et al., 2016; Steele et al., 2018). The correlation of MST markers with pathogen presence in environmental samples has provided mixed results in MST field studies (Tambalo et al., 2012; Marti et al., 2013). Field studies to evaluate the correlation of MST marker and pathogens presence in a watershed are important for the assessment of the associated public health risk, have not previously been carried out in the Taihu watershed region.

Taihu Lake, one of the top five freshwater lakes in China, serves as a very important source of water supply, aquaculture, tourism, flood protection, and transportation. The Taihu watershed, located in southeast Jiangsu province and the Yangtze River basin, is one of the most populated regions of China, with a population density of 1,200 people per km^2^ and annual production of approximately six million pigs and/or chickens (Qin et al., 2007). It is reported that decline in the water quality of Taihu Lake is due to pollutants such as untreated sewage, animal and industrial waste inputs from rivers associated with the volume of river discharge entering the lake (Zhang Y. et al., 2016). Inputs from rivers are the main route of contaminants and nutrients, to the detriment of the ecological health of the lake (Saxena et al., 2015). Currently, more than 200 rivers are connected to Taihu Lake but there are only 13 primary inflow rivers (Zhang Y. et al., 2016). Tiaoxi River contributes approximately 60% of the total source water to Taihu Lake, significantly driving the water quality status of Taihu Lake (Zhang Y. et al., 2016).

Here, we report the results of a comprehensive evaluation of existing general and host-specific MST qPCR markers, using fecal samples collected from the Taihu watershed region, in order to identify the most suitable MST qPCR assays for detecting host associated fecal pollution across this large and important water catchment. Secondly, this study reports the presence and abundance of MST and pathogenic bacterial gene markers in the Tiaoxi River water and sediment samples, in order to assess the fecal contamination of this river and to characterize correlations across the FIB, MST, and specific bacterial pathogens markers.

## Materials and Methods

### Overview of the Study Area

The mainstream of the Tiaoxi River is 158 km in length consisting of the Eastern, Western, Southern, and Northern Tiaoxi Rivers (Zhang Y. et al., 2016). The Tiaoxi River flows in northern Zhejiang province covering the upstream agricultural areas and the downstream urban cities of the northern Zhejiang Province. It has been estimated that the river collects water from one million inhabitants residing in the moderately sized cities of Zhejiang Province (Zhang Y. et al., 2016). Poultry is a common livestock in Zhejiang province, and waste disposal is an issue (Zheijiang, 2016). Excluding poultry, pigs comprise more than 90% of the remaining livestock population in this region (NBSC, 2015). It has been reported that the Tiaoxi River is severely polluted by various sources such as farmlands, domestic sewage, and industrial waste, subsequently affecting the Taihu Lake water quality (Hagedorn and Liang, 2011; Lv et al., 2015).

### Evaluation of Existing MST Markers for Applicability in Taihu Watershed

#### Selection of MST qPCR Assays and Fecal Sampling

The preliminary selection of MST assays for this study was based on the identity of the livestock population in Zhejiang Province and as per the data released by the National Bureau of Statistics, PR China in 2015 (NBSC, 2015). The MST assays developed and designed elsewhere were selected to validate their applicability; the details of the MST assays are provided in Supplementary Table S1. To determine the specificity and sensitivity of host-associated MST qPCR markers, individual and composite fecal and wastewater samples were used as target sources (Ahmed et al., 2016b). In total, 61 fresh individual and composite fecal samples, collected from various hosts in Huzhou (Zhejiang province) and Suzhou (Jiangsu province), were tested. The details of the collection of fecal and wastewater samples and preparation of composite fecal samples are provided in Supplementary Note S1. Individual fecal samples from animal hosts representing pigs, chickens, dogs, and cows (*n* = 10 for each) and composite fecal sources from ducks and geese (*n* = 3 pooled samples, each pooled sample was prepared from five individual fecal samples) were collected from farms, pet stores and the backyards of households located near the Taihu Lake/Tiaoxi River watershed region in the Huzhou area. All fecal samples were transported to the laboratory on ice and stored at -20°C within 6 h of collection. Primary effluents (500 ml; *n* = 6) were collected from a wastewater treatment plant (WWTP) located in Suzhou and brought to the laboratory on ice. Biomass from primary influents was collected by centrifugation at 4,000 × *g* for 10 min at 4°C, and DNA was extracted immediately. Ethical approval for handling fecal samples in this study was acquired from Xi’an Jiaotong-Liverpool University (XJTLU) Research Ethics Committee.

#### DNA Extraction and qPCR Assay Conditions

DNA extraction from the fecal/sewage samples was performed using the PowerFecal^®^ DNA isolation kit that uses Inhibitor Removal Technology^®^ (IRT) (MoBio, Carlsbad, CA, United States), following the manufacturer’s instructions. The quality and quantity of extracted DNA was confirmed by NanoDrop ND 2000C (Thermo Fisher Scientific., United States) and extracts were stored at -20°C prior to further analysis. Further quality assurance of extracted DNA samples and the details of plasmid standard construction for MST qPCR assays are provided in Supplementary Note S2.

All qPCR reactions (20 μl) were performed in triplicate and the reaction mixture for all Taqman chemistry-based qPCR assays included 10 μl of TaqMan Environmental Master Mix 2.0 (Applied Biosystems, Foster City, CA, United States), 2 μl of the probe/primer set with a final concentration as shown in Supplementary Table S1 and 8 μl of the 10-fold diluted target DNA template. For the two SYBR Green chemistry based qPCR assays (HF183 SYBR and GFD), the reaction mixture contained 10 μl of SYBR Green Master Mix 2.0 (Thermo Scientific, United States), 2 μl of primer mixture and 8 μl of the 10-fold diluted target DNA templates as stated above. The correct amplification products for these SYBR Green assays were chosen based on the melting curve analysis as described previously (Seurinck et al., 2005; Green et al., 2012).

#### qPCR Performance Characteristics and Data Analysis

For each MST assay, the limit of detection (LOD) was determined from the standard curve. The lowest concentration of standard gene copies that could be confidently detected in all triplicates was considered as the LOD (Schriewer et al., 2013; Ahmed et al., 2016a). All the qPCR results were normalized to gene copies/ng of DNA and the samples considered positive if the concentrations were above the LOD. The interpretation of the qPCR assay results was as stated in the previous studies (Ahmed et al., 2016b; Nshimyimana et al., 2017). Sensitivity and specificity of all qPCR assays were determined as described previously (Ahmed et al., 2013). The statistical and qPCR data analyses were carried out using either Microsoft Excel or SPSS version 22.0. The linear regression analysis was performed using Microsoft Excel; the statistical significance in the abundance of Bac-Uni and GenBac3 markers of fecal and sewage samples was determined using SPSS 22.0 (IBM Inc., Chicago, IL, United States).

### Quantification of MST and Pathogenic Bacterial Gene Markers in Tiaoxi River Water and Sediment Samples

#### Sample Collection and Processing

**FIGURE 1:**
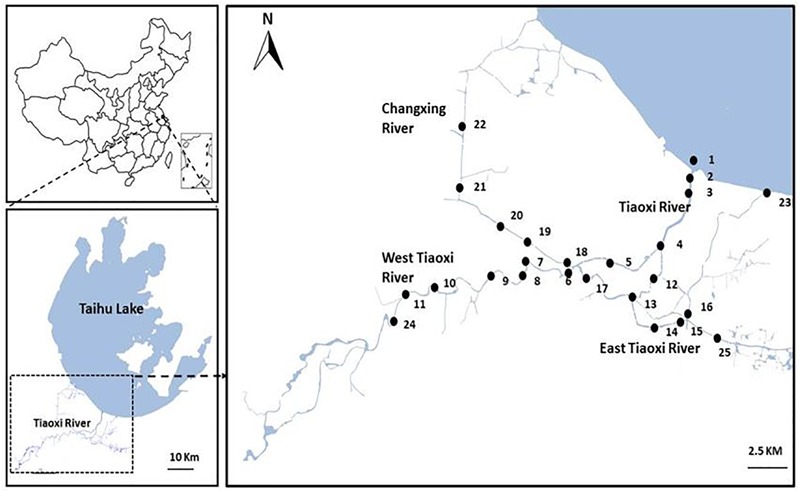
Map of the sampling locations selected for the current study in Tiaoxi River (Taihu watershed).

Initially, 25 sampling locations were selected covering the East and West, and junctions of the Tiaoxi River with other tributaries, extending to approximately 100 km of the mainstream river (Figure 1); samples were collected from these locations in three seasons (autumn 2014, winter, and summer 2015). Locations comprising domestic, agricultural, and industrial areas were selected for sampling and the land use pattern of the sampling locations was reported in our previous study (Vadde et al., 2018); the details are also provided in Supplementary Table S2. Water samples were collected in sterile 5 L polypropylene containers and sediments were collected using a sediment sampler; the samples in triplicate were transferred to sterile 50 ml tubes. Water and sediment samples were transported to the laboratory on ice and were processed within 8 h. Sediment samples were frozen at -20°C and the water samples (250 ml) were filtered through 0.22 μm polycarbonate membrane filters (Millipore, United Kingdom) and stored at -20°C prior to DNA extraction. The DNA was extracted from membrane filters (water samples) and sediment samples using PowerSoil DNA Isolation Kit (MoBio Inc., Carlsbad, CA, United States) as per the manufacturer’s instructions.

#### Enumeration of Fecal Coliforms

The current study incorporates microbiological assessment data published in our previous study (Vadde et al., 2018). The enumeration of fecal coliforms (FC) was carried out by standard membrane filtration technique (APHA, 2005) using mFC agar (Difco, Germany) according to the manufacturer’s instructions. The FC count data were used for initial assessment of fecal pollution at the Tiaoxi River and to prioritize the locations for further quantification of MST and pathogenic bacterial gene markers at these locations.

#### Quantification of MST and Pathogenic Bacterial Gene Markers by qPCR

As validated in this study, four previously designed qPCR assays targeting total, human, swine, and avian associated fecal sources were selected for their quantification in water and sediment samples of the Tiaoxi River (Table 1). Three TaqMan assays (BacUni, HF183 Taqman, and Pig-2-Bac) were selected for detection of the total, human and swine associated *Bacteroidale*s, and one SYBR green-GFD assay was selected for detection of avian associated fecal markers (Kildare et al., 2007; Mieszkin et al., 2009; Green et al., 2012, 2014). In addition, five qPCR assays targeting *stx2, eae, LipL32, ipaH*, and *mapA* genes of the specific pathogenic bacteria were selected for this study (Table 1). Two qPCR assays targeting the *eae* gene specific for *E. coli* O157:H7 (Ibekwe et al., 2002) and *stx2* gene specific for Shiga toxin producing *E. coli* (Beutin et al., 2008) were applied using SYBR Green chemistry. Three assays targeting the *LipL32* gene specific for pathogenic *Leptospira* sp. (Stoddard et al., 2009), *ipaH* gene specific for *Shigella* spp. (Wang et al., 2007) and *mapA* gene specific for *Campylobacter jejuni* (Best et al., 2003) used TaqMan chemistry.

**Table 1:** List of primers and probes used for quantification of MST markers and genes of pathogenic bacteria.

Target source/					Annealing
organism	Assay	Primer/probe	Concentration	Oligonucleotide sequence (5′–3′)	temperature (°C)	References
Total *Bacteroidales*	BacUni	BacUni-520F	400 nM	CGTTATCCGGATTTATTGGGTTTA	60	Kildare et al., 2007
		BacUni-690R1	400 nM	CAATCGGAGTTCTTCGTGATATCTA		
		BacUni656P	80 nM	FAM-TGGTGTAGCGGTGAAA-MGB		
Human associated *Bacteroidales*	HF183	HF183F	1000 nM	ATCATGAGTTCACATGTCCG	60	Green et al., 2014
		BacR287R	1000 nM	CTTCCTCTCAGAACCCCTATCC		
		BacP234P	80 nM	FAM-CTAATGGAACGCATCCC-MGB		
Swine associated *Bacteroidales*	Pig-2-Bac	Pig-2-Bac41F	300 nM	GCATGAATTTAGCTTGCTAAATTTGAT	60	Mieszkin et al., 2009
		Pig-2-Bac163R	300 nM	ACCTCATACGGTATTAATCCGC		
		Pig-2-Bac113P	200 nM	VIC-TCCACGGGATAGCC-MGB		
Avian associated Marker	GFD	GFD-F	100 nM	TCGGCTGAGCACTCTAGGG	57	Green et al., 2012
		GFD-R	100 nM	GCGTCTCTTTGTACATCCCA		
*C. jejuni*	*mapA*	mapA F	400 nM	CTGGTGGTTTTGAAGCAAAGATT	60	Best et al., 2003
		mapA R	400 nM	CAATACCAGTGTCTAAAGTGCGTTTAT		
		mapA P	80 nM	FAM-TTGAATTCCAACATCGCTAATGTATA AAAGCCCTTT-TAMRA		
Pathogenic *Leptospira* spp.	*LipL32*	LipL32F	300 nM	AAG CAT TAC CGC TTG TGG TG	60	Stoddard et al., 2009
		LipL32R	300 nM	GAA CTC CCA TTT CAG CGA TT		
		LipL32P	200 nM	FAM-AAAGCCAGGACAAGCGCCG-BHQ1		
*Shigella* spp.	*ipaH*	ipaH F	400 nM	CTTGACCGCCTTTCCGATA	64	Oster et al., 2014
		ipaH R	400 nM	AGCGAAAGACTGCTGTCGAAG-		
		ipaH P	80 nM	FAM-AAC AGG TCG CTG CAT GGC TGG AA-TAMRA		
*E. coli* (STEC)	*stx2*	Stx2F	200 nM	CAGGCAGATACAGAGAGAATTTCG	61	Beutin et al., 2008
		Stx2R	200 nM	CCGGCGTCATCGTATACACA		
*E. coli* O157:H7	*eae*	eae-F	200 nM	GTAAGTTACACTATAAAAGCACCGTCG	56	Ibekwe et al., 2002
		eae-R	200 nM	TCTGTGTGGATGGTAATAAATTTTTG		

All qPCR reactions were run in triplicate with a final reaction volume of 20 μL. A seven-point 10-fold serially diluted recombinant plasmid DNA with a target sequence was used to generate the standard curve (range 10^1^ to 10^7^ copies/reaction) in each qPCR assay. All the TaqMan qPCR assays (20 μl of master mix), contained 10 μl of TaqMan Environmental Master Mix 2.0 (Applied Biosystems, United Kingdom), 2 μL of template DNA, 6 μl nuclease-free water and 2 μL of primers and probes at the final concentrations shown in Table 1. SYBR Green assays (20 μl of master mix) contained 10 μl of SYBR Green PCR Master Mix (Thermo Fisher Technologies, Foster City, CA, United States), 7.0 μL nuclease-free water, 2 μL of template DNA and 1 μL of primer mixture with a final concentration as in Table 1. Prior to quantification, the absence of PCR inhibitors was analyzed in at least 10% of DNA samples by applying the BacUni qPCR assay that amplifies the 16S rRNA gene of *Bacteroidales* (Odagiri et al., 2015). The accuracy and efficiency of the standard curves were determined by including a positive control of 10^3^ copies of the plasmid standard as the unknown in each assay (Oster et al., 2014).

#### Data Processing and Statistical Analyses

All the qPCR assay results were processed based on the Minimum Information for Publication of Quantitative Real-Time PCR Experiments (MIQE) guidelines (Bustin et al., 2009); if the *R*^2^ or efficiencies were not achieved by any assay, the samples were tested again. The details of the LOD, limit of quantification (LOQ), and final assessment of qPCR results for each of the MST and pathogen quantification assays are provided in Supplementary Information. For statistical analysis, the concentrations of FC, MST markers, and genes of pathogenic bacteria were log transformed and non-detects were assigned as 0 (Bradshaw et al., 2016). The data were not normally distributed (based on Kolmogorov–Smirnov test) after transformation, therefore Kruskal–Wallis non-parametric ANOVA with Dunn’s post-test was used for determining statistical significance among different sampling locations. The correlation among FC, MST markers and genes of pathogenic bacteria in water samples was analyzed by Spearman’s coefficient correlation.

## Results

### Evaluation of Existing MST Makers for Applicability in Taihu Watershed

#### Quality Assurance of Fecal DNA and Performance Characteristics of MST qPCR Assays

Reliable quantification and specific detection of genetic markers by qPCR poses many challenges, therefore assays should be carefully designed and optimized to obtain maximum achievable specificity and sensitivity (Bustin et al., 2009). The quality assurance of fecal/sewage DNA was assessed by the BacUni assay and the results showed that all the fecal/sewage DNA had matching concentrations (based on mean Cp and percentage of coefficient of variation) of BacUni markers in the two different dilutions tested (1:10 and 1:100), indicating the absence or negligible amounts of PCR inhibitors. Therefore, all further assays were performed with 1:10 diluted DNA extracts. The Cp mean values for the BacUni marker in 1:10 and 1:100 diluted human fecal samples are provided in Supplementary Table S3.

The amplification efficiencies of all MST qPCR assays tested were in the range 86–102% and the correlation coefficient (*r*^2^) values were ≥0.98. The detailed performance characteristics of all the qPCR assays are provided in the Supplementary Table S4; all of the values were within the limits recommended in the MIQE guidelines (Bustin et al., 2009). All of the qPCR standards were re-analyzed to determine the master standard curve with standardized slope, amplification efficiencies, and correlation coefficient (*r*^2^) values. The details of master standard curves and LOD for each tested MST assay are presented in Table 2.

**Table 2:** Performance characteristics of MST qPCR assays tested using fecal and sewage samples.

Assay	Slope	y-intercept	*R*^2^	Efficiency	LOD^∗^ (gc/rxn)
BacUni	-3.32	43.3	0.99	100.0	24.5
GenBac3	-3.27	40.3	0.99	101.8	14.1
BacHum	-3.26	38.4	0.99	102	36.8
HF183 Taqman	-3.37	39.6	0.99	99.7	11.5
HumM2	-3.34	42.2	0.99	98.9	75
HF183 SYBR	-3.3	36.2	0.99	100.9	10
Pig-2-Bac	-3.27	41.1	0.99	102	30
BacCow	-3.31	41.6	0.99	100.3	100
AV4143	-3.5	43	0.99	93	10
GFD	-3.41	36.9	0.99	96	11.3

*^∗^LOD, limit of detection.*

#### Performance of Universal and Human *Bacteroidales* Assays

Both BacUni and GenBac3 assays, targeting universal/general *Bacteroidales*, exhibited 100% sensitivity to fecal and sewage samples as they amplified DNA from all the samples. The mean concentration of these markers in fecal and sewage samples is given in Supplementary Table S5. Comparatively, BacUni showed slightly higher total *Bacteroidales* concentrations [copies per nanogram (ng) of DNA] than the GenBac3 assay in all of the tested samples.

Among the four human-associated assays tested, the HF183 SYBR marker was the most sensitive but showed high cross-reactivity (Table 3). It was found in 80% of human origin samples (7/10 human feces and 5/5 sewage samples) at an average concentration of 2.72 log_10_ gene copies per ng of DNA and showed cross reactivity with chicken (7/10), cow (3/10), duck (1/3), dog (2/10), and goose (1/3) fecal DNA samples identifying it as the least specific (69.5%). The BacHum marker had the highest specificity (80.4%) among the tested human-associated markers, along with HF183 Taqman. The BacHum assay had a sensitivity of 73.3% (6/10 human feces and 5/5 sewage samples) at an average concentration of 3.19 log_10_ copies per ng of DNA and was detected (above LOD) in three different host fecal DNA samples – chicken (6/10), cow (1/10), and dog (2/10). Similar to BacHum, the HF183 Taqman marker was highly specific (80.4%) among human-associated assays and was found in 73.3% of target sources (6/10 human feces and 5/5 sewage samples) at an average concentration of 3.63 log_10_ copies per ng of DNA. However, HF183 Taqman marker was found in only two different host fecal DNA samples, chicken (7/10) and dog (2/10). The final human-associated assay, Hum2 was the least sensitive marker and was detected in only 53% of samples of human origin (7/10 human feces and 1/5 sewage samples) at an average concentration of 2.52 log_10_ copies per ng of DNA. It was also detected in chicken (4/10), cow (2/10), pig (2/10), dog (1/10), and goose (1/3) fecal DNA samples, making it less specific (78%). In general, chicken (40–70%) and dog fecal samples (10–20%) had cross-reactivity with all of the human-associated assays. Overall, HF183 Taqman was the only assay that did not exhibit any cross-reactivity with cattle or swine fecal DNA samples, while HF183 SYBR, Hum2, and BacHum showed cross-reactivity with either cattle or swine fecal DNA samples (Table 3).

**Table 3:** Performance of human-associated *Bacteroidales* MST assays on fecal and sewage samples.

	No. of
	samples
Source	tested	BacHum		HF183 Taqman		HF183 SYBR		Hum2	
		No. of positive	Mean (±SD^a^) concentration	No. of positive	Mean (±SD) concentration	No. of positive	Mean (±SD) concentration	No. of positive	Mean (±SD) concentration
		samples (%)	(log_10_ gene copies per ng)	samples (%)	(log_10_ gene copies per ng)	samples (%)	(log_10_ gene copies per ng)	samples (%)	(log_10_ gene copies per ng)
Human	10	6 (60)	3.98 (±0.75)	6 (60)	4.51 (±0.78)	7 (70)	3.99 (±0.81)	7 (70)	3.28 (±0.31)
Sewage	5	5 (100)	2.41 (±0.69)	5 (100)	2.73 (±0.25)	5 (100)	1.46 (±0.72)	1 (20)	1.76
Pig	10	0 (0)	0	0 (0)	0	0	0	2 (20)	2.46 (±1.20)
Chicken	10	6 (60)	2.53 (±0.37)	7 (70)	3.22 (±0.3)	7 (70)	2.18 (±0.45)	4 (40)	2.03 (±0.73)
Cow	10	1 (10)	1.8	0 (0)	0	3 (30)	1.38 (±0.48)	2 (20)	2.12 (±0.93)
Dog	10	2 (20)	1.75 (±0.47)	2 (20)	1.36 (0.52)	2 (20)	1.43 (±0.12)	1 (10)	2.24
Duck^∗^	3	0 (0)	0	0 (0)	0	1 (33)	1.41	0 (0)	0
Goose^∗^	3	0 (0)	0	0 (0)	0	1 (33)	1.68	1 (33)	1.97
Target			3.19 (±1.11)		3.63 (±1.25)		2.72 (±0.93)		2.52 (±1.07)
Non-target			2.14 (±1.07)		2.5 (±1.31)		1.62 (±0.33)		2.16 (±0.19)
Sensitivity		73.3%		73.3%		80%		53.3%	
Specificity		80.4%		80.4%		69.5%		78.2%	

*^∗^Composite fecal samples. ^*a*^SD, standard deviation.*

#### Performance of Animal Associated MST Assays

The performance of the swine associated assay (Pig-2-Bac) was evaluated with 10 pig fecal samples and the target was found in 90% of samples (9/10) at an average concentration of 3.03 log_10_ copies per ng of DNA (Table 4). The Pig-2-Bac marker was highly specific (95%) and it had a low level of cross-reactivity with cow fecal samples (2/10). BacCow marker was found in 100% of cattle fecal samples (10/10) at an average concentration of 2.81 log_10_ copies per ng of DNA (Table 4), but showed some cross-reactivity with pig (2/10) and chicken (2/10) fecal DNA samples, although not found in any of the human fecal DNA samples at above LOD levels, yielding a specificity value of 77%.

**Table 4:** Performance of animal associated MST assays on fecal samples.

	No. of
	samples
Source	tested	Pig-2-Bac		BacCow		AV4143		GFD	
		No. of positive	Mean (±SD) concentration	No. of positive	Mean (±SD) concentration	No. of positive	Mean (±SD) concentration	No. of positive	Mean (±SD) concentration
		samples (%)	(log_10_ gene copies per ng)	samples (%)	(log_10_ gene copies per ng)	samples (%)	(log_10_ gene copies per ng)	samples (%)	(log_10_ gene copies per ng)
Human	10	0 (0)	0	0 (0)	0	1 (10)	1.7	0 (0)	0
Pig	10	9 (90)	3.03 (±0.59)	2 (20)	2.52 (±0.89)	0 (0)	0	0 (0)	0
Chicken	10	0 (0)	0	2 (20)	2.32 (±0.77)	10 (100)	4.13 (±0.49)	7 (70)	2.20 (±0.19)
Cow	10	2 (20)	1.70 (±0.84)	10 (100)	4.3 (±0.65)	2 (20)	1.3 (±086)	1 (10)	1.2
Dog	10	0 (0)	0	0 (0)	0	0 (0)	0	3 (30)	1.3 (±0.40)
Target			3.03 (±0.59)		4.30 (±0.65)		4.13 (±0.49)		2.20 (±0.19)
Non-target			1.70 (±0.84)		2.42 (±0.14)		1.5 (±0.2)		1.25 (±0.07)
Sensitivity		90%		100%		100%		70%	
Specificity		95%		77.5%		95%		92.50%	

The avian associated MST assays performed very distinctively on the tested fecal DNA samples (Table 4). The AV4143 marker was found in 100% of chicken fecal samples but was also detected in human (1/10) and cow (2/10) fecal DNA. The mean concentration of AV4143 marker was 4.13 log_10_ copies per ng of DNA in chicken feces. Those human and cow samples for which there was cross reactivity comprised ca. 3 log-fold fewer gene copies than chicken feces. The GFD markers were only detected in 7 of the 10 chicken feces samples and cross-reacted with dog fecal DNA samples (3/10). The mean concentration of GFD markers was 2.20 log_10_ copies per ng of fecal DNA sample, so ca. 100-fold less than AV4143.

### Assessment of Microbial Quality of Tiaoxi River Water and Sediments

#### Enumeration of Fecal Coliforms in Water Samples

**FIGURE 2:**
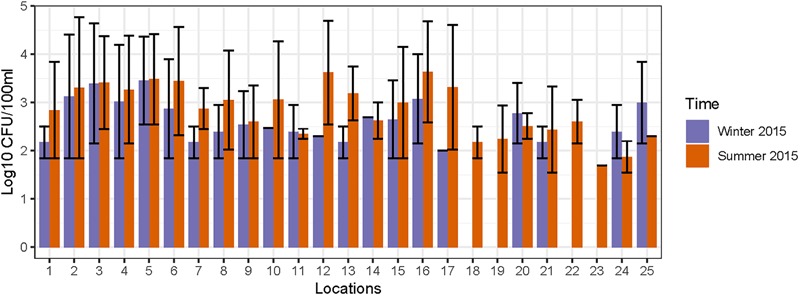
Concentration of fecal coliforms observed in different sampling locations of Tiaoxi River.

FC count data was used for an initial assessment of fecal contamination at these sampling locations and the results reported in our previous study (Vadde et al., 2018). The FC counts are interpreted according to US EPA standards (USEPA, 2012), and elevated levels of FC (>250 CFU/100 ml) were detected at 15 locations (1–6, 8, 10, 12–16, 20, and 21) on one or more occasions (Figure 2). In general, the FC counts were higher during summer, which could be correlated with the warm (optimal) weather supporting acclimatization of FC bacteria; a rainfall event that occurred the day before the sampling may have led to transport of fecal matter and proliferation of FC bacteria (Heaney et al., 2015). Based on the elevated levels of FC, 15 out of 25 locations were considered as preliminary hotspots of fecal contamination and were selected for further quantification of MST fecal markers and pathogenic bacterial genes in DNA extracted from water and sediment samples. The FC counts (range and mean) along with physico-chemical analysis results for 25 sampling locations monitored during 2014 to 2015 is provided in Supplementary Table S6.

#### Quality Assurance of Extracted DNA and Performance of qPCR Assays

DNA samples tested for PCR inhibition did not show any major difference in concentration values (based on mean Cp and percentage of coefficient of variation) for undiluted and 1:4 diluted DNA extracts, indicating that the samples were free of potential PCR inhibitors. The range and compiled values of amplification efficiencies and linear range of quantification for all qPCR assays are provided in Supplementary Table S7. The average Cp value for all negative controls (NTC) was >38, and samples were repeat tested if the average Cp value of NTCs was <38.

#### Detection Frequency and Concentration of MST Markers in Water and Sediment Samples

**FIGURE 3:**
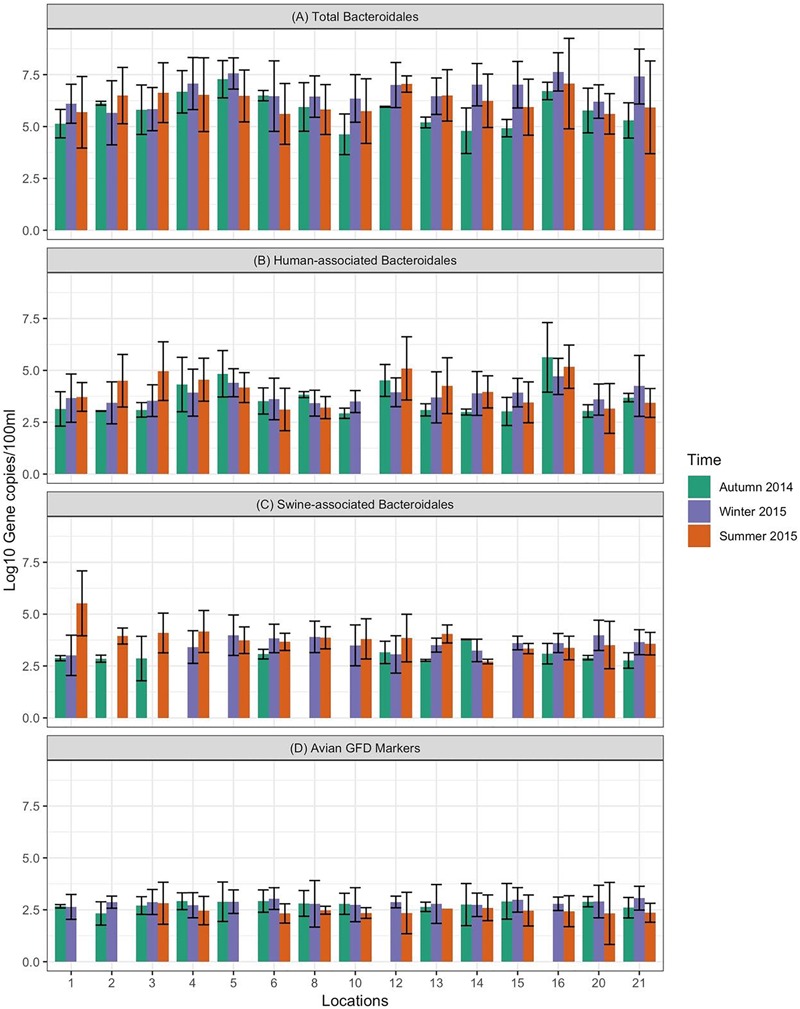
Concentration of MST fecal markers quantified in water samples at different sampling locations of Tiaoxi River. **(A)** Total *Bacteroidales*; **(B)** human-associated *Bacteroidales*; **(C)** swine-associated *Bacteroidales*, and **(D)** avian-associated MST marker.

**FIGURE 4:**
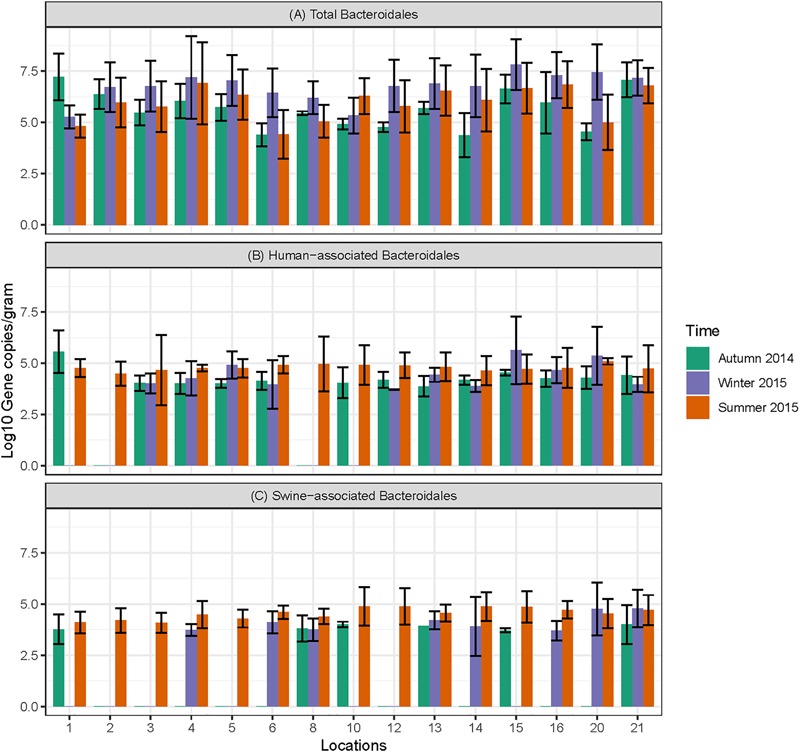
Concentration of MST markers quantified in sediment samples at different sampling locations in Tiaoxi River. **(A)** Total *Bacteroidales*; **(B)** human-associated *Bacteroidales*; **(C)** swine-associated *Bacteroidales*.

Data on the presence and distribution of MST markers in water and sediment samples at 15 sampling locations, determined by qPCR, are presented in Table 5. Since these monitoring locations selected for the MST study were presumed hot spots of fecal contamination (based on FC count), the total *Bacteroidales* marker was detected in all water and sediment samples at the 15 locations. In water samples, the mean concentration of total *Bacteroidales* marker was 6.22 log_10_ gene copies/100 ml with concentrations ranging from 4.62 to 7.63 log_10_ gene copies/100 ml (Figure 3A). For sediment, the mean concentration was 6.11 log_10_ gene copies/gram and concentrations ranged from 4.37 to 7.82 log_10_ gene copies/gram (Figure 4A). Significant statistical variation in total *Bacteroidales* concentrations among different locations was observed for both water (*P* > 0.015) and sediment samples (*P* > 0.003). In relative terms, the total *Bacteroidales* concentration was high during winter for both water and sediment samples. Based on the quantification of total *Bacteroidales*, the water samples from location 16 and sediment samples from location 15 were found to be the most fecally polluted, regardless of the fecal source (human versus animal species). Locations 15 and 16 are suburban areas, which are close to a WWTP. For the host associated MST marker analysis in water samples, human-associated markers were the most frequently detected (97%), followed by avian (89%) and swine (84%). In sediment samples, human-associated MST marker was detected more often (86%), followed by swine (64%). The avian markers were positive for several sediment samples but were always below the LOD.

**Table 5:** Detection frequencies of MST markers in water and sediment samples of Tiaoxi River, Taihu watershed (2014–2015).

	No. of
Sample	samples
type	tested (*n*)	No. of positive samples (%)^a^			
			Human	Swine	Avian
		Total	associated	associated	associated
		*Bacteroidales*	markers	markers	markers
**Water**					
Autumn	15	15 (100%)	15 (100%)	10 (66%)	13 (86%)
Winter	15	15 (100%)	15 (100%)	13 (86%)	15 (100%)
Summer	15	15 (100%)	14 (93%)	15 (100%)	13 (86%)
**Total**	**45**	**45 (100%)**	**44 (97%)**	**38 (84%)**	**40 (89%)**
**Sediment**					
Autumn	15	15 (100%)	13 (86%)	6 (40%)	–*^b^*
Winter	15	15 (100%)	11 (73%)	8 (53%)	–*^b^*
Summer	15	15 (100%)	15 (100%)	15 (100%)	4 (26.7%)
**Total**	**45**	**45 (100%)**	**39 (86%)**	**29 (64%)**	**4 (8.9%)**

*^*a*^ Limit of detection (LOD) as cutoff. ^*b*^ Below detection limits.*

The human-associated marker, HF183 Taqman, was detectable at most of the locations tested in water samples (44/45) in all three seasons with a mean concentration of 3.75 log_10_ gene copies/100 ml (range 2.91–5.6 log_10_ gene copies/100 ml) (Figure 3B). For sediment samples, the HF183 Taqman was detected with high frequency (39/45) at concentrations ranging from 3.8 to 5.6 log_10_ gene copies/gram (mean 3.91 log_10_ gene copies/gram) (Figure 4B). During the summer, human-associated markers were detected at a higher prevalence in water and sediment samples, probably due to the runoff water received from heavy rainfall (349 and 268 mm in July and August 2015) that occurred before summer sampling (NBSC, 2016). There was a statistically significant difference in the prevalence of human markers in the sediment samples (*P* > 0.004) but not in the water samples. Based on the HF183 Taqman assay, water samples collected from locations 12 and 16, and sediment samples collected from location 15 had the highest concentration of human-associated marker during the three seasons. Location 12 is very close to Huzhou city and as stated above, locations 15 and 16 are suburban areas close to a WWTP, and the sampling was carried out at the junction of Tiaoxi River and a canal that enters Taihu Lake (Vadde et al., 2018) (Supplementary Table S2).

With respect to swine fecal contamination, the swine associated marker was detected less often in water (38/45) and sediment samples (29/45) than human markers. The mean concentration of swine marker in water samples was 2.96 log_10_ gene copies/100 ml (range 2.77–5.56 log_10_ gene copies/100 ml) (Figure 3C). The mean concentration of swine markers in sediment samples was 3.75 log_10_ gene copies/gram (range 3.6–4.7 log_10_ gene copies/gram) (Figure 4C). The variation in the concentration of swine markers detected in water (*P* > 0.001) and sediment samples (*P* > 0.001) was statistically significant. Higher concentrations of swine associated marker were observed in water samples at location 1 during summer and in sediment samples at location 10 during winter. The samples at location 1 were collected 1 km inside the Taihu Lake from the junction with the Tiaoxi River, and runoff during the summer was probably a contributory factor. Location 10 was close to a rural area where the agricultural input to the river upstream was likely to enhance the swine marker content.

The avian associated fecal pollution was found to be the second dominant host associated fecal pollution in the Tiaoxi River water samples. The avian markers were detected in 89% of water samples (40/45) at concentrations of 2.30–5.56 log_10_ gene copies/100 ml (mean 2.70 log_10_ gene copies/100 ml) (Figure 3D). There was no significant statistical variation for avian marker concentration in water samples across different locations of the study area. For sediment samples, the avian markers were detected in only four of the 15 samples collected in the summer season. During the autumn and winter seasons, although some samples were positive for the avian MST assay, they were below the LOD limits. The highest avian marker concentration was observed at location 6 for both water and sediment samples. Location 6 is in a suburban area near Qijia village, where several swine and poultry farms are located.

#### Concentration of Pathogen Bacterial Genes in Water and Sediment Samples

**FIGURE 5:**
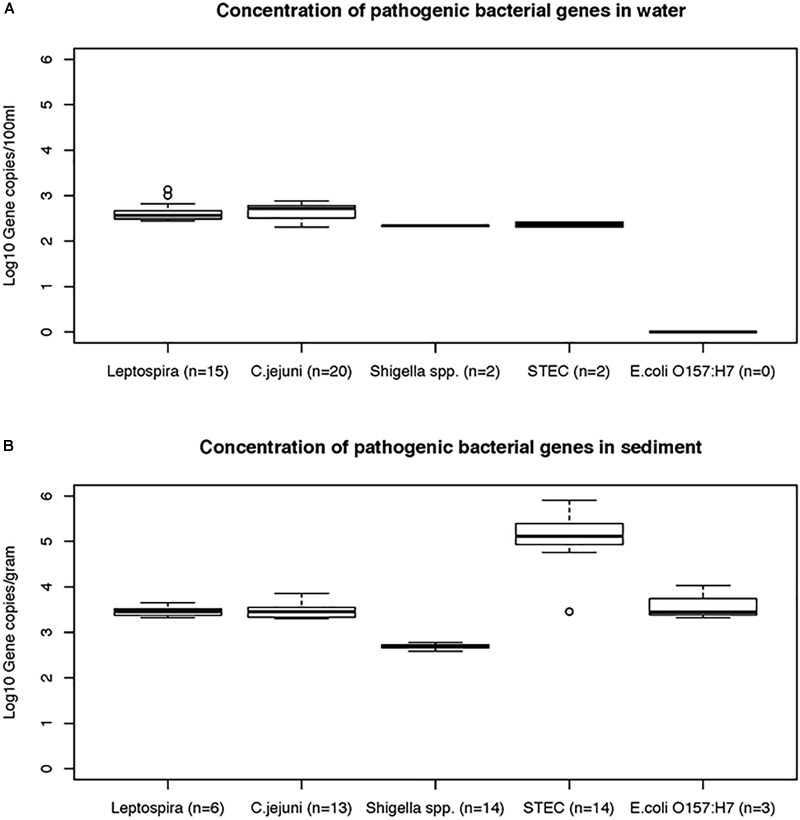
The concentrations (mean and standard deviations) of genes of bacterial pathogens detected in water **(A)** and sediment samples **(B)** of Tiaoxi River.

Data on the presence and distribution of gene markers for five bacterial pathogens are presented in Table 6. Considering detected but not quantifiable (DNQs) as positive samples, the most commonly detected pathogens in water and sediment samples, respectively, were *Campylobacter jejuni* (62% and 53%) and *Shigella* spp. (60% and 91%), followed by STEC (55% and 51%) and pathogenic *Leptospira* spp. (33% and 13%). The *E. coli* O157:H7 marker was detected only in sediment samples (11%). The concentrations of marker genes of bacterial pathogens detected in water and sediment samples at each location are provided in Supplementary Table S8. Using the LOQ as the selection criterion, *Campylobacter jejuni* (*mapA*) was present in a quantifiable range in 20 out of 45 water samples (2.31–2.88 log_10_ gene copies/100 ml) and 13 of 45 sediment samples (3.30–3.86 log_10_ gene copies/gram) (Figure 5). The highest *mapA* gene concentration was observed in water samples collected at location 16, and in sediment samples collected at location 20 (Supplementary Table S8). In the case of *Shigella* spp., although the *ipaH* gene was detected in several samples, it was quantifiable in only two out of 45 water samples (locations 3 and 12) with concentrations of 2.32 and 2.35 log_10_ gene copies/100 ml, respectively. In sediments, the *ipaH* gene was quantified in 14 samples with concentrations ranging 3.32–3.47 log_10_ gene copies/gram (Figure 5) and the highest concentration was observed at location 12. Similarly, the *stx2* (Shiga toxin-producing *E. coli*) was quantified in only 2 out of 45 water samples (locations 5 and 21) with a concentration of 2.31 and 2.42 log_10_ gene copies/100 ml, respectively. In sediments, it was quantified in 14 out of 45 samples with a concentration range of 3.32–3.65 log_10_ gene copies/gram (Figure 5) and the highest concentration of *stx2* genes was observed at location 14. The *LipL32* gene (Pathogenic *Leptospira* spp.) was quantified in 15 out of 45 water samples with the concentration ranging from 2.43 to 3.13 log_10_ gene copies/100 ml, with the highest levels recorded at location 21. The *LipL32* gene was quantified in 6 (out of 45) sediment samples at a concentration of 3.32–3.65 log_10_ gene copies/gram (Figure 5) and highest concentrations were observed at location 20. The *eae* gene of *E. coli* O157:H7 was quantified in only three sediment samples collected in autumn 2014 with a concentration of 3.32–4.03 log_10_ gene copies/gram and the highest concentration recorded at location 20 (Supplementary Table S8).

**Table 6:** Detection frequencies of bacterial pathogen marker genes in water and sediment samples of Tiaoxi River, Taihu watershed (2014–2015).

Sample type	No. of samples
	tested (*n*)	No. of positive samples*^a^*				
		*Leptospira*	*Campylobacter*	*Shigella*	*STEC*	*EHEC O157:H7*
		(*LipL32*)	(*mapA*)	(*ipaH*)	(*stx2*)	(*eae*)
**Water**						
Autumn	15	5 (33%)	4 (26.6)	15 (100%)	0	0
Winter	15	10 (66%)	9 (60%)	8 (53%)	12 (80%)	0
Summer	15	0	15 (100%)	4 (26.6)	12 (80%)	0
**Total**	**45**	**15 (33%)**	**28 (62%)**	**27 (60%)**	**25 (55%)**	**0**
**Sediment**						
Autumn	15	0	6 (40%)	15 (100%)	11 (73%)	5 (33%)
Winter	15	6	10 (66%)	12 (80%)	3 (20%)	0
Summer	15	0	8 (53%)	14 (97%)	9 (60%)	0
**Total**	**45**	**6 (13%)**	**24 (53%)**	**41 (91%)**	**23 (51%)**	**5 (11%)**

*^*a*^ DNQ’s (detected but not quantifiable) recorded as positive samples.*

#### Correlation Between FC, MST Markers, and Genes of Bacterial Pathogens

All the correlations in this study were considered as significant only when Rho(*r*) and *p-*values were >0.5 and <0.05, respectively (Steele et al., 2018). The concentrations of FC (FIB) were positively correlated with BacUni (*r* = 0.667 and *p* < 0.01) and HF183 Taqman (*r* = 0.572 and *p* < 0.05) but not with Pig-2-Bac and GFD markers (Table 7), indicating that the common source of fecal pollution is effluents from WWTP or human waste entry into the river. The FC did not show correlation with any of the genes of bacterial pathogens tested in this study, which is in agreement with previous studies (Bradshaw et al., 2016; Zhang Q. et al., 2016); FC points to the potential risk of exposure to pathogens but does not demonstrate their specific presence. The BacUni marker showed a strong positive correlation with HF183 Taqman (*r* = 0.832 and *p* < 0.01), suggesting humans as the major contributor to the total *Bacteroidales* content. BacUni also showed positive correlation with *stx2* gene (*r* = 0.6 and *p* < 0.05). HF183 Taqman showed negative correlation with GFD marker (*r* = -0.582 and *p* < 0.05) and positive correlation with *stx2* gene (*r* = 0.593 and *p* < 0.05). Since there are multiple fecal sources (such as pig, cow, and poultry) of *stx2* gene presence in the environment, it is difficult to draw conclusions on the *stx2* gene correlation with BacUni and HF183 Taqman. No significant correlation was observed for Pig-2-Bac and GFD markers with the marker genes of bacterial pathogens addressed in this study.

**Table 7:** Correlation between FC, MST markers and pathogenic bacterial marker genes.

	Correlation coefficient								
	FC	BacUni	HF183Taq	Pig-2-Bac	GFD	*LipL32*	*mapA*	*ipaH*	*stx2*
FC	1	0.667**	0.572*	-0.234	-0.27	-0.248	0.095	-0.361	0.397
BacUni		1	0.832**	-0.036	-0.396	-0.04	-0.018	-0.168	0.600*
HF183Taq			1	-0.061	-0.582*	-0.265	0.1	-0.121	0.593*
Pig-2-Bac				1	-0.268	0.346	0.5	0.068	-0.421
GFD					1	-0.095	0	0.057	-0.275
*LipL32*						1	-0.251	-0.225	0.207
*mapA*							1	0.379	-0.286
*ipaH*								1	-0.018
*stx2*									1

*^∗∗^Correlation is significant at 0.01 level (two-tailed). ^∗^Correlation is significant at 0.05 level (two-tailed).*

## Discussion

### Evaluation of MST qPCR Assays for Their Applicability at Taihu Lake/Tiaoxi River Watershed

Overall, this is a comprehensive study evaluating the performance of existing universal, human and animal associated MST qPCR assays for their applicability to ascertain host-associated fecal pollution in the Taihu Lake/Tiaoxi River watershed, China.

Among universal/total *Bacteroidales* MST qPCR assays, the high sensitivity of the BacUni (Universal *Bacteroidales*) marker for human and animal feces, excluding avian sources, has frequently been reported in Asian countries such as India (Odagiri et al., 2015) and Singapore (Nshimyimana et al., 2017), and also in the United States (Kildare et al., 2007), and Kenya (Jenkins et al., 2009). The GenBac3 general *Bacteroidales* assay is the most widely used assay in the United States for quantifying *Bacteroidales* in environmental samples (Shanks et al., 2009; Ervin et al., 2013). In our study, both BacUni and GenBac3 showed 100% sensitivity to fecal samples but the BacUni assay amplified higher copies per ng of DNA than the GenBac3 assay in all of the tested samples. We conclude that the BacUni assay is more suitable for quantification of total *Bacteroidales* in the Taihu watershed, China; Odagiri et al. (2015) also reported that the BacUni marker quantified higher copy numbers than GenBac3 in fecal DNA samples tested in Odisha, India. The ability of human-associated markers to identify human fecal sources as distinct from other sources in an aquatic environment is vital to the MST approach. Variations, due for example to the impact of dietary patterns on gut microbiomes (Wu et al., 2011) or geographical differences (Yatsunenko et al., 2012), could significantly affect the performance of MST assays and this has been supported by several validation studies across different countries in recent years (Jenkins et al., 2009; Reischer et al., 2013; Odagiri et al., 2015; Boehm et al., 2016; He et al., 2016; Nshimyimana et al., 2017; Malla et al., 2018). Therefore, it is important to evaluate the performance of human fecal markers prior to application. Here, with the exception of the HF183 SYBR assay, all of the human-associated assays showed lower sensitivity (53–73%) than expected (81–100%). This reduced sensitivity range could well be due to the geographical variability in human gut microbiomes (Yatsunenko et al., 2012). The HF183 SYBR assay, which performed well in Bangladesh (Ahmed et al., 2010) and in Singapore as a human sewage indicator (Nshimyimana et al., 2014), showed less specificity in our study. Our results on the specificity of this assay are more in line with previous studies conducted in India (Odagiri et al., 2015) and Nepal (Malla et al., 2018). Although the remaining three assays are less sensitive compared to HF183 SYBR, they are more specific. The BacHum and HF183 Taqman assays showed relatively more specificity (80.4%), followed by Hum2 with 72.5% specificity. All of the human target assays gave some level of cross-reactivity with other fecal samples, with the highest levels recorded in chickens (Table 3). The high cross-reactivity with chicken fecal samples by human-associated markers has been described in earlier studies directed in South Asia (Odagiri et al., 2015; Nshimyimana et al., 2017). The occurrence of the HF183 marker in dogs and chickens has also been reported previously, with the HF183 forward primers with Bac708 reverse primer picking up a target sequence in chicken fecal samples (Gourmelon et al., 2007; Ahmed et al., 2012). The BacHum assay’s cross-reactivity with chicken fecal DNA has also been indicated previously in several studies (Reischer et al., 2013; Odagiri et al., 2015; Nshimyimana et al., 2017). With the exception of HumM2, none of the markers exhibited cross-reactivity with pig fecal DNA, which is encouraging as pigs are major livestock animals in Zhejiang province (Taihu watershed region). An assay that showed zero cross-reactivity to pig fecal DNA and highly sensitive to sewage samples would be considered as very suitable for source tracking in the Taihu watershed region. In this study, HF183 Taqman and BacHum assays showed the same accuracy, but HF183 Taqman was found to be the more suitable human-associated fecal maker than BacHum as it amplified DNA from all of the sewage samples with higher abundance and did not show any cross-reactivity with pig and cattle fecal samples (Table 3). The HF183 Taqman’s cross-reactivity to chicken fecal samples can be negated by employing avian associated assays, such as GFD assay, in tandem to verify the existence of true human fecal pollution. An evaluation study carried out in Singapore (Nshimyimana et al., 2017) indicated similar sensitivity and specificity for HF183 Taqman to that reported here, though that study recommended another human-associated assay, B. theta (Taqman), for source tracking of human fecal and sewage in Singapore. The inconsistencies in the performance of *Bacteroidales* assays in regions other than the originally developed have been reported elsewhere (Layton et al., 2013; Reischer et al., 2013; Boehm et al., 2016).

The swine associated marker, Pig-2-Bac, has broadly been evaluated and used for MST studies across different countries, including China and Nepal (Jia et al., 2014; Arfken et al., 2015; He et al., 2016; Malla et al., 2018). He et al. (2016) validated the Pig-2-Bac assay on target and non-target fecal samples of Taihu watershed region and reported that it was more sensitive and specific than mitochondrial DNA based swine markers. They have also applied the Pig-2-Bac marker for identifying swine fecal pollution in the Taige River of Taihu watershed. Jia et al. (2014) used Pig-2-Bac assay for quantification of swine fecal markers in Hongqi River, Yongan River, and Taige River. The results of the swine associated assay in our study are in agreement with the findings of He et al. (2016), and the suitability of the Pig-2-Bac assay for identifying contamination by pig fecal sources at Taihu watershed is confirmed. The BacCow marker, which was originally developed to detect fecal source of cow or cattle origin (Kildare et al., 2007), has shown cross-reactivity with fecal samples collected from other ruminants (e.g., deer) and non-ruminants such as horse, pig, dog, and chicken in a California based validation study leading to its reclassification as ruminant-associated marker (Raith et al., 2013). Similar findings were reported in evaluation studies conducted in Australia and Europe, indicating that this assay had cross-reactivity with non-targets such as chicken, goose, dog, pig, and duck (Ahmed et al., 2013; Reischer et al., 2013). In a validation study conducted in India, BacCow markers were reported to be found in all types of composite livestock/domestic animal feces (cow, buffalo, goat, sheep, dog, and chicken) but not in tested human samples and it was recommended that the BacCow assay could be used to detect fecal pollution by livestock/domestic animals (Odagiri et al., 2015). More recently, BacCow was detected in all fecal samples tested including human origin (composite sewage) in a validation study conducted in Nepal (Malla et al., 2018). In order to check the true specificity and sensitivity of BacCow, this study was conducted with individual fecal samples instead of composite fecal samples. Here, we detected BacCow in all of the cattle fecal samples tested but found some cross-reactivity with pig (2/10) and chicken (2/10) samples in agreement with the findings of Ahmed et al. (2013) and Reischer et al. (2013). Since the BacCow marker was not identified in all of the livestock/domestic fecal samples tested, it is not applicable in the Taihu watershed as a livestock/domestic animal fecal source-tracking marker.

The ability to draw conclusions on the microbial flora in avian fecal samples is still ambiguous worldwide, making it difficult to develop reliable qPCR assays for the specific detection of avian fecal contaminations (Ohad et al., 2016). This could be due to variation in food intake by the avian sources regionally (Ahmed et al., 2016a). Though *Bacteroides* and its closely related organisms are commonly used for identifying the source of fecal contamination for human and animals (Bernhard and Field, 2000; Layton et al., 2006; Kildare et al., 2007), previous studies revealed that *Bacteroides* are rarely present in avian sources (Lu et al., 2009). The phylum level mapping of avian fecal samples showed that Firmicutes, Proteobacteria, and Fusobacteria are the main phyla (Lu et al., 2009). *Bacteroidales* members were not frequently reported in avian gut or excreta, and they were found to be nearly absent in a few studies (Zhu et al., 2002) or they were identified in varying frequencies in other studies (Scupham et al., 2008). Therefore, MST markers targeting avian fecal markers are still limited (Fremaux et al., 2010; Green et al., 2012; Ohad et al., 2016). We found that the AV4143 assay targeting *Lactobacillus* showed higher sensitivity and specificity than the GFD assay targeting *Helicobacter* spp. However, GFD markers were not detected in human fecal samples making this assay very suitable for application in the Taihu watershed as it can reliably differentiate chicken and human fecal samples. The GFD assay was similarly validated in Australia, Bangladesh, New Zealand, and North America with similar results for sensitivity and specificity (Ahmed et al., 2016b; Boehm et al., 2016). Overall, BacUni, HF183 Taqman, Pig-2-Bac, and GFD markers are recommended here, for tracking total and host-associated fecal contamination in the Taihu watershed region. The results also reinforce the importance of regional MST validation studies prior to their application in a new geographical region.

### Application of Evaluated MST qPCR Assays to Track the Source of Fecal Pollution in Tiaoxi River

Fecal pollution of surface waters is a serious concern to the aquatic ecosystem and human health. In this study, fecal coliform counts were higher than suggested limits at 15 out of 25 monitoring locations, and increased concentrations were observed during the summer season. Acclimatization of existing FC bacteria to warm temperatures or entry of fresh feces from different sources such as human, animal or sewage into the surface water due to runoff from rainfall event occurred prior to sampling could have elevated the levels of FC during summer (Sidhu et al., 2012). Therefore, MST was conducted to assess the presence of fresh fecal pollution in the Tiaoxi River and to determine the fecal sources at these locations. In general, the distribution of MST markers among different locations correlated well with the land use pattern and results indicated that there was a mixed input of fecal pollution at several locations. Presence of Total *Bacteroidales* markers with high frequency in water and sediment samples at all fifteen locations (Figure 3A, 4A) shows possible fresh fecal source entry and transportation to other locations within the study area (Marti et al., 2013). Human-associated markers were consistently identified (in both water and sediment) in most of the sampling locations during three occasions (Figure 3B, 4B). Except for summer season (July/August 2015) where a rainfall event (∼349/268 mm) occurred, autumn (October 2014) and winter (January/February 2015) were dry seasons (with only 32 and 66 mm precipitation) indicating that fecal contamination at these locations might not be merely through runoff but could be due to direct discharge of sewer and septic waste (Ohad et al., 2015). The detection of *Shigella* spp. that solely originate from human fecal sources at several locations also points to human fecal contamination in the studied area. High levels of human-associated markers were observed in water samples at locations 3, 5, 12, and 16 and in sediment samples at location 15 on one or more occasions. The higher levels of human-associated markers observed in water samples at locations 5 and 16 and sediment samples at 15 could be associated with effluents from WWTP located near these locations (Zheng et al., 2017). The higher levels of human-associated markers in water samples at location 3 (rural area) and 12 (urban area) that does not have agricultural or effluent entry from WWTP (Vadde et al., 2018) (Supplementary Table S2), indicates that the major source of human-associated markers at these locations could be sewage (Kapoor et al., 2015).

Swine farming is the dominant livestock-based agricultural activity in Zhejiang province. The swine associated marker was frequently detected in water samples collected at locations 6, 12, 13, 20, and 21 and sediment samples collected in locations 13 and 21 (Figure 3C, 4C). The results obtained were consistent with the land use pattern as these locations have active pig-farming operations. Location 6 is near Qijia village where commercial and household backyard pig and poultry farms were observed during the sampling. On the upstream side of location 13, WWTP and active pig farming (Supplementary Table S9) were found near to this location (Zheng et al., 2017). Locations 20 and 21 were close to Changxing port, which has several farms for pigs and poultry (Supplementary Table S7). The only possible explanation for the consistent detection of swine associated markers at location 12 is the transport of bacteria from location 13, as the distance between two locations is ∼1 km. In China, both backyard and commercial based poultry farming are common and the existence of such farms nearby leads to the release of poultry feces into the watershed (Zhuang et al., 2017). The provincial government of Zhejiang had concerns over the illegal discharge of poultry wastes into the watershed and has recently initiated monitoring control measures (Zheijiang, 2016). The avian associated marker quantification results prove that poultry fecal pollution was high in the study area as markers were detected in 89% of water samples (40/45) (Figure 3D). Based on the GFD assay, water samples collected at locations 6, 15, 20, and 21 (Figure 3D) and sediments (summer season) collected at locations 6 and 20 (data not shown here) had high levels of avian markers. Active commercial and backyard poultry farming was observed at these locations (locations 6, 20, and 21) and results correlate well with the land use pattern (Supplementary Tables S2, S9) (Vadde et al., 2018). Although the avian associated marker quantification results were positive for sediment samples collected from several locations in autumn and winter, the quantities were below the detection limit, i.e., LOD. Overall, the human associated *Bacteroidales* MST marker was the most frequently detected host-associated MST marker in the sediment samples, highlighting the presence of human fecal pollution in the Tiaoxi River. The survival and persistence of *Bacteroidales* associated MST markers in sediment samples, which could act as a non-point source of fecal pollution, has previously been reported (Kim and Wuertz, 2015). However, recent studies have reported that the HF183 Taqman marker showed limited persistence (<7 days) in freshwater sediments and suggested that it could be a reliable marker to detect recent fecal input into freshwater sediment, although these studies also cautioned that the decay of this marker in a different geographical setting could be influenced by various factors such as organic matter and nutrients (Zimmer-Faust et al., 2017; Ahmed et al., 2019). Overall, MST results obtained in the present study indicate the potential occurrence of pathogens at these locations, which was followed up with in-depth monitoring of host-associated pathogens.

### Detection of Pathogenic Bacterial Gene Markers to Assess the Potential Health Risk Posed by Tiaoxi River

Although MST is a promising technique for identifying the source of fecal contamination, a combination of a fecal indicator organism enumeration, MST, and associated waterborne pathogenic bacterial gene marker quantification, provides more reliable information on water quality and associated health risks in the watershed. Such studies provide valuable information to water quality management authorities, taking tangible actions to reduce pollution in contaminated waters.

Among the bacterial pathogens monitored, *Campylobacter jejuni* was found to be the most frequently detected pathogen in the study area (Table 6). *C. jejuni* originates primarily from chicken and other avian feces (Lund et al., 2004) and although high frequency of *C. jejuni* detection could be indicative of high avian fecal contamination, this organism can survive for up to 4 months in the environment (Murphy et al., 2006). *C. jejuni* was frequently detected in water and sediment samples at locations 4, 6, 8, and 13 and the concentrations were higher at locations 6 and 13 on one or more occasions, indicating that these locations could pose significant health risks. *Campylobacter jejuni* infective doses for human gastroenteritis have been reported to be as low as 500 cells (Ahmed et al., 2009). Globally, *Shigella* spp. are among the major bacterial causes of diarrhea; they are considered as one of the top four groups of pathogens that causes moderate to severe diarrhea in the children of Africa and South Asia (Huynh et al., 2015). *Shigella sonnei* is the most commonly recovered species in infected patients of the United States, while in Asia and developing countries, *S. dysenteriae* and *S. flexneri* were the major causative species (Wu et al., 2009). In the present study, the *Shigella* marker gene, *ipaH*, was detected in 68 (75.6%) of the 90 water and sediment samples tested (Table 6). Humans are considered as the common and natural reservoirs for *Shigella* spp. (Ishii et al., 2013) and the presence of these bacteria indicates human fecal contamination (Oster et al., 2014). As the *ipaH* gene occurs in 5–10 copies in plasmid and genomic DNA of *Shigella* spp. (Greenberg et al., 2010), only *Shigella* spp. detected in water and sediment samples at locations 3 and 12 were found to be at higher concentrations and may pose a potential human health risk. Monitoring of *Shigella* and *Campylobacter* spp. is a useful tool for watershed managers/monitoring agencies as these organisms are associated with specific sources (host associated). Shiga toxin-producing *E. coli* (STEC) can cause gastrointestinal disease leading to mild or severe bloody diarrhea (Haack et al., 2015) and they originate from multiple sources or reservoirs (Beutin et al., 2008). Here, although STEC was detected in nearly 50% of water samples tested in the study area (Table 6), only locations 5 and 21 had higher concentrations indicating significant human health risk at these locations. Pathogenic *Leptospira* species cause leptospirosis by colonization of the renal tubules of infected reservoir hosts such as dogs, rats and, cattle, and leptospires enter the aquatic environment via urine (Monahan et al., 2008). Pathogenic *Leptospira* spp. have a high infective dose due to the acid sensitivity of the bacteria (Ganoza et al., 2010). Therefore, the pathogenic *Leptospira* quantified in the present study may not be of significant human health risks, although the higher concentrations were frequently detected at locations 20 and 21. *E. coli* O157:H7 (*eae* gene) was not detected in any of the water samples, but detected at low frequency (33%) in sediment samples (during autumn season) (Figure 5). It has been reported that cattle are the main reservoir for *E. coli* O157:H7 (Ahmed et al., 2009) and the results presented here are consistent with the paucity of cattle farming in the watershed area (NBSC, 2015). Overall, the quantification of the genes of bacterial pathogens in water and sediment samples indicate that *C. jejuni* and STEC are the major concerns for human health risk in a few specific locations of the study area. Furthermore, the bacterial pathogen quantification results correlate with the findings of host associated fecal markers, demonstrating the potential of MST in predicting the presence of pathogenic organisms and the concomitant risk to human health.

## Conclusion

In summary, our MST qPCR validation study demonstrates that BacUni, HF183 Taqman, Pig-2-Bac, and GFD assays are the most suitable for differentially identifying and monitoring human and animal fecal contamination in the Taihu watershed. In the case of fecal pollution monitoring at the Tiaoxi River, 15 out of 25 monitoring locations were identified as hotspots of fecal contamination based on FC enumeration, and samples collected from those 15 locations were further used for the quantification of MST and pathogenic bacterial gene markers. The total *Bacteroidales* marker was detected in all of the water and sediment samples at these 15 monitoring locations, confirming the presence of fecal contamination. Although human-associated markers were frequently detected at several locations, locations 3, 12, and 16 had high concentrations on one or more occasions, indicating that they are major human fecal contaminated sites of the Tiaoxi River region. The swine associated marker was frequently detected in samples from locations 13 and 21 and the avian associated marker was detected with high concentrations at locations 6, 15, 20, and 21, matching the land use pattern and pointing to the entry of swine and avian fecal sources to the Tiaoxi River. Among five bacterial pathogens monitored, *Campylobacter jejuni* was frequently detected at locations that are primarily polluted with avian fecal material (locations 4, 6, 8, and 13) and high concentrations were detected at locations 6 and 13 (on one or more occasions) indicating that these sites pose potential human health risks. Similarly, *Shigella* spp. were frequently detected at higher concentrations in two locations (locations 3 and 12) that are highly contaminated with human fecal sources, and the Shiga toxin-producing *E. coli* (STEC) at two locations (locations 5 and 21), which are contaminated with either human or pig fecal sources. The bacterial pathogen quantification results correlate with the findings of host-associated fecal markers, and the data generated here are valuable for water quality monitoring authorities responsible for minimizing the health risks associated with pathogens, by identifying sites where intervention is required.

## Ethics Statement

Ethical approval for this study was provided by XJTLU Research Ethics Committee (EXT-16-07-01).

## Author Contributions

RS, AM, and RR conceived and designed the experiments. RS and KV carried out the field sampling. KV performed the experiments and data analyses and prepared the manuscript with the directions of his supervisors RS, AM, and RR. RS and AM contributed to the revision of the manuscript.

## Conflict of Interest Statement

The authors declare that the research was conducted in the absence of any commercial or financial relationships that could be construed as a potential conflict of interest.
