# Supplementary material for: Quantification of Microbial Source Tracking and Pathogenic Bacterial Markers in Water and Sediments of Tiaoxi River (Taihu Watershed)
Source: Front Microbiol. 2019 Apr 24;10:699. doi: 10.3389/fmicb.2019.00699 (PMC6492492; doi:10.3389/fmicb.2019.00699)
Supplement: Supplementary file 1 [file Table_1.docx]

Supplementary Material

Quantification of microbial source tracking and pathogenic bacterial markers in water and sediments of Tiaoxi River (Taihu watershed)

Kiran Kumar Vadde^1^, Alan J. McCarthy^2^, Rong Rong^1^ and Raju Sekar^1^*

^1^Department of Biological Sciences, Xi’an Jiaotong-Liverpool University, Suzhou 215123, China.

^2^Microbiology Research Group, Institute of Integrative Biology, University of Liverpool, Liverpool L69 7ZB, United Kingdom.

*** Correspondence:** Raju Sekar: [Sekar.Raju@xjtlu.edu.cn](mailto:Sekar.Raju@xjtlu.edu.cn)

**Supplementary Note S1.** **Fecal sampling and composite sample preparation:**

Fresh fecal droppings from animals were collected in sterile containers. Individual pig fecal samples were collected from pig farms located at Changxing Zhicheng (N30°59′26.53″; E119°53′24.67″) and Wuxing Daishan (N30°53′24.92″; E120°11′59.15″) areas of Zhejiang province. Individual dog fecal samples were collected from different pet stores located in Hong Qiao (N31°33′45.68″; E120°20′20.19″) of Zhejiang province. Individual chicken, duck and goose samples were collected from poultry farms located at Cao Jian Duan Village (N30°54′9.94″; E120°18′40.81″), Baishui (N30°54′19.78″; E119°49′17.86″) and Lijiagang (N31°02′18.59″; E119°51′39.48″) areas of Zhejiang province. Individual cow fecal samples were collected from slaughterhouses at Changxing Zhicheng (N30°59′26.53″; E119°53′24.67″) of Zhejiang Province. All fresh fecal droppings from animals were collected in sterile containers. Fresh human fecal samples were collected in sterile containers from healthy volunteers (n=10) aged between 16 and 40 years. Safety guidelines were provided and consent for use of the samples in this study was obtained. Ethical approval was acquired from XJTLU Research Ethics Committee for handling fecal and sewage samples in this study. Individual human fecal samples were 10 different individuals of age 16-40 years. Primary effluents were collected on six different occasions from a WWTP located in Suzhou (N31°17′37.54″   E120°34′10.00″), Jiangsu province. For preparation of composites for duck and goose samples, approximately 0.5gms of individual fecal samples (>5 samples) of a host (goose or duck) were pooled together to form a composite sample of respective host. The composite samples were used for further DNA extractions.

**Table S1.** List of primers and probes used for validation of MST qPCR assays in samples collected from the Taihu watershed region

| **Assay** | **Primer/probe** | **Concentration** | **Oligonucleotide sequence (5’–3’)** | **Annealing temperature (^o^C)** | | **Reference** |
| --- | --- | --- | --- | --- | --- | --- |
| BacUni (Taqman) | BacUni- 520F  BacUni-690R1 BacUni-690R2  BacUni656P | 400nM  400nM  400nM  80nM | CGTTATCCGGATTTATTGGGTTTA CAATCGGAGTTCTTCGTGATATCTA  AATCGGAGTTCCTCGTGATATCTA  FAM-TGGTGTAGCGGTGAAA-MGB | | 60 | (Kildare et al., 2007) |
| GenBac3  (Taqman) | GenBacF3  GenBac4R  GenBact2P | 1000nM  1000nM  80nM | GGGGTTCTGAGAGGAAGGT  CCGTCATCCTTCACGCTACT  FAM-CAATATTCCTCACTGCTGCCTCCCGTA-TAMRA | | 60 | (Siefring et al., 2008) |
| HF183 (TaqMan) | HF183F  BacR287R  BacP234P | 1000nM  1000nM  80nM | ATCATGAGTTCACATGTCCG  CTTCCTCTCAGAACCCCTATCC  FAM-CTAATGGAACGCATCCC-MGB | | 60 | (Green et al., 2014) |
| BacHum (Taqman) | BacHum-160F  BacHum-241R  BacHum-193P | 400nM  400nM  80nM | TGAGTTCACATGTCCGCATGA CGTTACCCCGCCTACTATCTAATG  6-FAM-TCCGGTAGACGATGGGGATGCGTT-TAMRA | | 60 | (Kildare et al., 2007) |
| HF183 (SYBR Green) | HF183F  Bac242R | 100nM  100nM | ATCATGAGTTCACATGTCCG  TACCCCGCCTACTATCTAATG | | 53 | (Seurinck et al., 2005) |
| HumM2 (Taqman) | Hum2F  Hum2R  HumM2P | 400nM  400nM  80nM | CGTCAGGTTTGTTTCGGTATTG  TCATCACGTAACTTATTTATATGCATTAGC  FAM-TATCGAAAATCTCACGGATTAACTCTTGTGTACGC-TAMRA | | 60 | (Shanks et al., 2009) |
| Pig-2-Bac  (Taqman) | Pig-2-Bac41F  Pig-2-Bac163R  Pig-2-Bac113P | 300nM  300nM  200nM | GCATGAATTTAGCTTGCTAAATTTGAT  ACCTCATACGGTATTAATCCGC  VIC-TCCACGGGATAGCC-MGB | | 60 | (Mieszkin et al., 2009) |
| BacCow  (Taqman) | BacCow-CF128F  BacCow-305R  BacCow-257P | 400nM  400nM  80nM | CCAACYTTCCCGWTACTC  GGACCGTGTCTCAGTTCCAGTG  6-FAM-TAGGGGTTCTGAGAGGAAGGTCCCCC- TAMRA | | 60 | (Kildare et al., 2007) |
| AV4143 (Taqman) | Av4143F  Av4143R  Av4143P | 500nM  500nM  250nM | TGCAAGTCGAACGAGGATTTCT  TCACCTTGGTAGGCCGTTACC  FAM-AGGTGGTTTTGCTATCGCTTT-BHQplus | | 60 | (Ohad et al., 2016) |
| GFD  (SYBR Green) | GFD-F  GFD-R | 100nM  100nM | TCGGCTGAGCACTCTAGGG  GCGTCTCTTTGTACATCCCA | | 57 | (Green et al., 2012) |

**Supplementary Note S2.**

**DNA extraction and quality assurance of extracted fecal/sewage DNA samples**

Approximately 250 mg of individual fecal/raw sewage samples were used for DNA extraction from all the host fecal sources except avian fecal samples for which only 0.10g was used. To avoid any cross contamination with other hosts, DNA extraction was conducted for each type of fecal source separately. Controls with no fecal samples were also performed simultaneously in each batch.

The DNA extracts were evaluated for the absence of PCR inhibitors and for the presence of amplifiable fecal DNA using the Bac-Uni qPCR assay (Odagiri et al., 2015) which detects universal *Bacteroidales* 16S rRNA genes. Two dilutions of DNA extracts (1:10 and 1:100) were assayed and the DNA extract was judged as free from PCR inhibitors if the two sample dilutions gave matching concentrations of Bac-Uni amplification products (Reischer et al., 2013;Odagiri et al., 2015).

**Preparation of DNA standards for qPCR assays**

Plasmid DNA standards were used for all qPCR assays and prepared by amplifying the target genes for each assay with the respective primer set using the fecal DNA extracts. The amplified products were purified using a PCR purification kit (Axygen Biosciences, CA, USA), ligated to pMD 19 vector (Takara, Bio Inc., Shiga, Japan) and transformed into competent *E. coli* cells (Tiangen Biotech, China). Plasmid DNA was extracted from the positive clones using QIAprep Spin Miniprep Kit (Qiagen, Mississauga, ON), and the extracted plasmids were sequenced using respective primers (Sangon Biotech, China). Plasmid DNA standards were quantified using NanoDrop ND 2000C spectrophotometer and these concentrations were used for calculation of gene copy numbers. Standard curves for each assay were developed using tenfold serial diluted plasmid standards containing the respective target gene (10^8^-10^1^ copies/µl for each reaction).

**Table S2.** Description of sampling locations along with coordinates and corresponding land use types (Vadde et al., 2018).

| **Sampling location** | **Description of location and land use types** | **Coordinates** |  |
| --- | --- | --- | --- |
|  |  | **Latitude** | **Longitude** |
| **1** | Taihu Lake and Tiaoxi River junction; 1 km inside the Taihu Lake; Aquaculture/fishing area. | N30°57′3.15″ | E120°07′42.64″ |
| **2** | Suburban area with aquaculture and fish handling/processing area. | N30°56′25.30″ | E120°07′35.72″ |
| **3** | Fishermen village; People live on boats stationed at this location. | N30°55′57.65″ | E120°07′37.27″ |
| **4** | Sub-urban area with residential apartments, businesses, and parks; East and West Tiaoxi River junction near south Taihu bridge. | N30°53′50.96″ | E120°06′0.95″ |
| **5** | Urban area with construction sites and various factories; Heavy ferry transportation were noticed in this area. Close to WWTP. | N30°53′19.40″ | E120°03′18.16″ |
| **6** | Suburban and industrial area with various factories; West Tiaoxi River and Changxin River junction. Active in Pig and Poultry farming. | N30°52′55.15″ | E120°0′58.87″ |
| **7** | Residential, farming and small industrial area close to a village; Various farm animals in small scale were noticed at the Riverbank. | N30°53′14.16″ | E119°58′38.58″ |
| **8** | Close to a town with businesses and residences, Ferry/boat docking area. Active in Pig and Poultry farming. | N30°53′1.82″ | E119°58′48.08″ |
| **9** | Rural agricultural area with sparse residential apartments. | N30°52′43.41″ | E119°56′43.37″ |
| **10** | Rural agriculture area with few industries (e.g. shipping industries and oil station); Heavy ferry transportation was noticed in this area. | N30°52′21.55″ | E119°53′55.85″ |
| **11** | Rural with a high number of residential apartments; Heavy ferry transportation was noticed in this area. | N30°52′8.11″ | E119°52′15.52″ |
| **12** | Urban area with businesses (e.g. many shopping malls) and residential apartments; Tourist boats docked close to this location. | N30°52′54.56″ | E120°06′1.47″ |
| **13** | Urban area with residential apartments and construction sites; Second junction between west and east Tiaoxi River. Pig and Poultry farms are present within 1km. | N30°51′56.74″ | E120°04′25.11″ |
| **14** | Sub-urban area with construction sites, residential apartments, and businesses; Ferry docking (large-scale) area. | N30°50′53.74″ | E120°05′38.57″ |
| **15** | Sub-urban area with residential apartments and businesses; Junction between east Tiaoxi and a small River that connects to Taihu; Sampled close to ferry docking (large-scale) area. | N30°50′59.27″ | E120°06′21.50″ |
| **16** | Sub-urban and residential/business area; Junction between the main River and a canal, which connects to Taihu Lake. | N30°51′27.75″ | E120°07′32.13″ |
| **17** | Sub-urban and sparse residential area; Sampled at the third junction between west and east Tiaoxi River. | N30°52′40.51″ | E120°01′58.88″ |
| **18** | Sub-urban and industrial area; Sampled in the junction of ChangXing and Tiaoxi River; Sampled near ferry docking station. | N30°53′11.17″ | E120°0′52.95″ |
| **19** | Rural/village, sparse residential and industrial area. | N30°54′2.88″ | E119°58′42.16″ |
| **20** | Rural/village and sparse residential/industrial area. Pig and Poultry farms are present near to this location. | N30°54′33.91″ | E119°57′31.34″ |
| **21** | Rural/village, residential and sparse industrial area. Pig and Poultry farms are present near to this location. | N30°55′52.05″ | E119°55′9.61″ |
| **22** | Rural/village and industrial area; Heavy ferry transportation; Sampled close to a factory and ferry docking station. | N30°57′45.22″ | E119°55′19.98″ |
| **23** | Rural/village area; Sampled in a small canal, which connects to Taihu Lake. | N30°55′53.87″ | E120°11′35.48″ |
| **24** | Rural/village and sparse residential /industrial area. | N30°51′0.12″ | E119°51′28.68″ |
| **25** | Sub-urban area with businesses and industries; Many small Rivers branch off from East Tiaoxi River. | N3050045.36” | E120°08′21.54″ |

**Table S3.** Confirmation of the absence of PCR inhibitors in human fecal samples with BacUni assay.

| **Samples** | **Ct value for 1:10 dilution** | **Ct value for 1:100 dilution** | **Coefficient of variation (CV) %** |
| --- | --- | --- | --- |
| H-1 | 18.32 | 22.30 | 0.41 |
| H-2 | 18.53 | 21.99 | 0.16 |
| H-3 | 17.84 | 22.04 | 0.52 |
| H-4 | 23.31 | 27.69 | 0.59 |
| H-5 | 16.04 | 20.02 | 0.41 |
| H-6 | 10.44 | 14.20 | 0.31 |
| H-7 | 13.41 | 17.27 | 0.36 |
| H-8 | 10.62 | 14.38 | 0.31 |
| H-9 | 12.26 | 16.29 | 0.44 |
| H-10 | 20.56 | 23.98 | 0.14 |

**Table S4.** Detailed performance characteristics (range) for all the qPCR assays.

| **Assay** | **Slope** | **R^2^ value** | **Efficiency (%)** |
| --- | --- | --- | --- |
| BacUni | -3.2 to -3.32 | 0.996 to 0.997 | 100.0 to 105.8 |
| GenBac | -3.26 to - 3.27 | 0.994 to 0.996 | 101.8 to 102.4 |
| HF183 Taqman | -3.32 to -3.35 | 0.996 to 0.999 | 98.16 to 99.7 |
| BacHum | -3.25 to -3.29 | 0.994 to 0.998 | 101 to 103.9 |
| HF183 SYBR | -3.26 to -3.30 | 0.997 to 0.999 | 100.7 to 102.3 |
| Hum2 | -3.22 to -3.36 | 0.994 to 0.996 | 98.4 to 105 |
| Pig-2-Bac | -3.27 to -3.35 | 0.997 to 0.998 | 98.5 to 102 |
| BacCow | -3.31 to -3.42 | 0.995 to 0.997 | 95.9 to 100.3 |
| AV4143 | -3.5 to -3.56 | 0.99 to 0.998 | 91.1 to 93.3 |
| GFD | -3.27 to 3.44 | 0.997 to 0.999 | 95.2 to 102 |

**Table S5.** Performance of universal/general *Bacteroidales* MST assays on fecal and sewage samples.

| **Source** | **No. of samples tested** | **Bac-Uni** | | **GenBac3** | |
| --- | --- | --- | --- | --- | --- |
|  |  | No. of positive  samples | Mean (±SD) Concentration  Log_10_ gene copies per ng | No. of positive  samples | Mean (±SD) Concentration  Log_10_ gene copies per ng |
|  |  |  |  |  |  |
| Human | 10 | 10 | 6.95 (0.41) | 10 | 5.91 (0.35) |
| Sewage | 5 | 5 | 5.36 (0.82) | 5 | 4.19 (1.30) |
| Pig | 10 | 10 | 5.39 (0.67) | 10 | 4.44 (0.79) |
| Chicken | 10 | 10 | 3.86 (0.12) | 10 | 2.85 (0.27) |
| Cow | 10 | 10 | 5.31 (0.90) | 10 | 4.80 (0.94) |
| Dog | 10 | 10 | 4.25 (0.32) | 10 | 3.82 (0.49) |
| Duck* | 3 | 3 | 4.12 (0.31) | - | - |
| Goose* | 3 | 3 | 5.81 (0.12) | - | - |
| Average |  |  | 5.19 (1.52) |  | 4.37 (1.56) |
| Sensitivity |  | 100% |  | 100% |  |

*****Composite fecal samples.

**Table S6.** Range, mean and standard deviation of different water quality parameters measured at different locations in Taihu watershed during 2014-2015.

| **Locations** | **TN (mg/L)** | | | **TP (µg/L)** | | | **NO_3_-N (mg/L)** | | | **NO_2_-N (mg/L)** | | | **PO_4_-P (µg/L)** | | |
| --- | --- | --- | --- | --- | --- | --- | --- | --- | --- | --- | --- | --- | --- | --- | --- |
|  | **Range** | **Mean** | **S.D** | **Range** | **Mean** | **S.D** | **Range** | **Mean** | **S.D** | **Range** | **Mean** | **S.D** | **Range** | **Mean** | **S.D** |
| Location 1 | 1.31-2.95 | 2.06 | 0.83 | 68.88-102.61 | 84.76 | 16.95 | 0.37-2.44 | 1.42 | 1.04 | 0.01-0.10 | 0.07 | 0.06 | 2.46-23.58 | 9.77 | 11.97 |
| Location 2 | 1.58-3.06 | 2.30 | 0.74 | 64.6-118.04 | 91.96 | 26.74 | 0.53-2.59 | 1.59 | 1.03 | 0.01-0.11 | 0.06 | 0.05 | 5.70-30/28 | 15.40 | 13.08 |
| Location 3 | 1.90-3.47 | 2.50 | 0.85 | 66.29-113.6 | 91.31 | 23.77 | 0.55-2.96 | 1.67 | 1.22 | 0.01-0.16 | 0.08 | 0.08 | 7.45-30.24 | 17.97 | 11.50 |
| Location 4 | 2.40-3.50 | 3.06 | 0.58 | 74.89-110.24 | 95.23 | 18.27 | 1.71-3.34 | 2.45 | 0.83 | 0.05-0.08 | 0.07 | 0.02 | 21.52-23.67 | 25.81 | 5.67 |
| Location 5 | 2.50-3.50 | 3.16 | 0.57 | 81.27-109.4 | 94.40 | 14.16 | 1.85-3.08 | 2.67 | 0.71 | 0.04-0.08 | 0.06 | 0.02 | 14.13-29.75 | 21.77 | 7.82 |
| Location 6 | 2.41-3.64 | 3.15 | 0.65 | 67.43-87.09 | 77.73 | 9.86 | 1.78-3.33 | 2.71 | 0.82 | 0.05-0.08 | 0.06 | 0.02 | 9.01-29.39 | 19.43 | 10.20 |
| Location 7 | 2.45-3.20 | 2.92 | 0.41 | 63.94-95.78 | 78.42 | 16.11 | 1.96-2.91 | 2.57 | 0.53 | 0.04-0.07 | 0.06 | 0.02 | 10.36-27.94 | 19.61 | 8.83 |
| Location 8 | 2.59-3.25 | 2.97 | 0.34 | 57.63-108.64 | 79.69 | 26.20 | 1.99-2.87 | 2.53 | 0.47 | 0.04-0.07 | 0.06 | 0.02 | 11.86-29.23 | 21.18 | 8.76 |
| Location 9 | 2.44-3.27 | 2.95 | 0.44 | 55.76-87.27 | 70.58 | 15.84 | 1.99-2.82 | 2.53 | 0.47 | 0.04-0.07 | 0.06 | 0.02 | 12.37-28.60 | 21.48 | 8.29 |
| Location 10 | 2.33-3.07 | 2.76 | 0.38 | 53.42-86.25 | 68.74 | 16.52 | 1.90-2.83 | 2.46 | 0.50 | 0.03-0.08 | 0.06 | 0.02 | 10.87-29.11 | 20.66 | 9.19 |
| Location 11 | 2.25-3.02 | 2.76 | 0.44 | 59.31-88.12 | 71.80 | 14.78 | 1.75-2.75 | 2.39 | 0.55 | 0.03-0.07 | 0.05 | 0.02 | 11.64-29.11 | 20.41 | 8.69 |
| Location 12 | 1.77-3.25 | 2.40 | 0.77 | 88.33-94.32 | 92.09 | 3.27 | 0.82-2.5 | 1.67 | 0.89 | 0.01-0.07 | 0.05 | 0.03 | 2.52-33.28 | 19.50 | 15.63 |
| Location 13 | 2.73-3.69 | 2.85 | 0.79 | 77.06-112.64 | 92.97 | 18.09 | 1.82-3.51 | 2.54 | 0.87 | 0.04-0.06 | 0.06 | 0.01 | 10.08-30.71 | 18.16 | 11.02 |
| Location 14 | 2.13-3.31 | 2.57 | 0.65 | 80.45-115.82 | 94.68 | 18.67 | 1.49-2.41 | 1.95 | 0.46 | 0.03-0.11 | 0.06 | 0.04 | 2.79-29.45 | 19.79 | 14.76 |
| Location 15 | 2.51-3.74 | 2.96 | 0.68 | 79.76-129.68 | 103.47 | 25.05 | 2.03-2.97 | 2.37 | 0.53 | 0.03-0.17 | 0.08 | 0.08 | 3.51-28.15 | 18.54 | 13.18 |
| Location 16 | 2.92-3.27 | 3.10 | 0.18 | 135.26-187.42 | 166.09 | 27.35 | 0.96-1.88 | 1.48 | 0.47 | 0.01-0.18 | 0.08 | 0.09 | 12.47-51.91 | 34.48 | 20.11 |
| Location 17 | 2.27-3.34 | 2.84 | 0.54 | 69.24-100.27 | 83.58 | 15.65 | 2.02-3.09 | 2.59 | 0.54 | 0.04-0.07 | 0.06 | 0.02 | 12.47-28.14 | 19.96 | 7.91 |
| Location 18 | 2.33-4.13 | 3.23 | 1.27 | 58.83-103.63 | 81.23 | 31.68 | 1.20-3.86 | 2.54 | 1.88 | 0.04-0.11 | 0.08 | 0.05 | 16.54-19.85 | 18.20 | 2.34 |
| Location 19 | 2.12-2.95 | 2.54 | 0.59 | 92.1-94.86 | 93.48 | 1.95 | 1.07-2.44 | 1.76 | 0.97 | 0.06-0.09 | 0.08 | 0.02 | 24.02-25.88 | 24.95 | 1.31 |
| Location 20 | 2.03-4.02 | 2.83 | 1.06 | 93.3-140.48 | 115.07 | 23.80 | 1.19-3.42 | 2.06 | 1.20 | 0.02-0.14 | 0.09 | 0.06 | 20.44-37.60 | 26.79 | 9.41 |
| Location 21 | 1.88-3.18 | 2.52 | 0.65 | 75.31-117.14 | 102.15 | 23.30 | 1.45-2.29 | 1.65 | 0.57 | 0.02-0.04 | 0.06 | 0.04 | 13.75-36.85 | 22.89 | 12.28 |
| Location 22 | 2.11-2.38 | 2.25 | 0.19 | 116.25-142.74 | 129.50 | 18.73 | 1.17-1.36 | 1.27 | 0.13 | 0.01-0.05 | 0.03 | 0.03 | 14.63-38.26 | 26.45 | 16.71 |
| Location 23 | 1.87-3.24 | 2.56 | 0.97 | 85.34-149.96 | 117.65 | 45.69 | 1.17-1.70 | 1.44 | 0.37 | 0.02-0.04 | 0.04 | 0.02 | 2.40-6.80 | 4.60 | 3.11 |
| Location 24 | 2.29-3.70 | 3.00 | 1.00 | 95.78-144.62 | 120.20 | 34.54 | 1.68-2.71 | 2.20 | 0.73 | 0.04-0.04 | 0.04 | 0.00 | 26.51-35.24 | 30.88 | 6.17 |
| Location 25 | 2.60-2.98 | 2.79 | 0.27 | 72.97-119.81 | 96.39 | 33.12 | 1.71-2.85 | 2.28 | 0.80 | 0.03-0.12 | 0.08 | 0.06 | 13.60-41.13 | 27.37 | 19.47 |

| **Locations** | **NH_4_-N (mg/L)** | | | **TOC (mg/L)** | | | **Chl *a* (µg/L)** | | | **TVC ( x 10^3^ CFU/mL)** | | | **TC ( x 10^2^ CFU/mL)** | | | **FC ( x 10^2^ CFU/100mL)** | | |
| --- | --- | --- | --- | --- | --- | --- | --- | --- | --- | --- | --- | --- | --- | --- | --- | --- | --- | --- |
|  | **Range** | **Mean** | **S.D** | **Range** | **Mean** | **S.D** | **Range** | **Mean** | **S.D** | **Range** | **Mean** | **S.D** | **Range** | **Mean** | **S.D** | **Range** | **Mean** | **S.D** |
| Location 1 | 0.05-0.06 | 0.08 | 0.03 | 3.83-8.45 | 5.44 | 2.62 | 80.3-102.3 | 91.33 | 11.1 | 1.15-15.50 | 8.15 | 7.18 | 2.25-12.40 | 7.66 | 5.11 | 1.50-7.00 | 4.25 | 3.89 |
| Location 2 | 0.11-0.24 | 0.18 | 0.06 | 2.44-12.11 | 7.48 | 4.85 | 75.9-95.9 | 85.87 | 10.0 | 3.74-10.00 | 5.98 | 3.49 | 2.50-20.40 | 10.30 | 9.17 | 13.50-20.50 | 17.00 | 4.95 |
| Location 3 | 0.06-0.27 | 0.20 | 0.11 | 2.27-15.81 | 8.47 | 6.84 | 47.3-87.5 | 70.23 | 20.7 | 7.05-11.80 | 8.88 | 2.56 | 5.00-21.33 | 12.91 | 8.18 | 25.00-26.00 | 25.50 | 0.71 |
| Location 4 | 0.04-0.66 | 0.32 | 0.32 | 2.75-16.71 | 8.70 | 7.21 | 40.7-86.7 | 66.67 | 23.6 | 6.12-8.65 | 7.46 | 1.27 | 10.55-13.67 | 11.66 | 1.75 | 10.50-18.50 | 14.50 | 5.66 |
| Location 5 | 0.06-0.57 | 0.30 | 0.26 | 1.97-14.81 | 7.79 | 6.51 | 53.9-83.2 | 69.90 | 14.8 | 4.19-22.05 | 14.01 | 9.06 | 10.50-20.75 | 17.08 | 5.71 | 28.50-30.50 | 29.50 | 1.41 |
| Location 6 | 0.12-0.43 | 0.25 | 0.16 | 3.67-11.31 | 5.79 | 4.83 | 53.9-77.4 | 68.33 | 12.6 | 3.04-9.80 | 6.95 | 3.50 | 1.00-9.50 | 6.05 | 4.47 | 7.50-28.00 | 17.75 | 14.5 |
| Location 7 | 0.11-0.44 | 0.26 | 0.17 | 4.00-11.31 | 5.25 | 3.29 | 37.4-53.4 | 43.83 | 8.5 | 5.43-9.00 | 6.84 | 1.90 | 1.50-15.00 | 6.67 | 7.29 | 1.50-7.50 | 4.50 | 4.24 |
| Location 8 | 0.12-0.46 | 0.27 | 0.18 | 2.75-10.51 | 5.58 | 4.28 | 37.4-59.8 | 47.07 | 11.5 | 6.45-8.00 | 7.21 | 0.78 | 1.50-12.33 | 6.19 | 5.56 | 2.50-11.25 | 6.88 | 6.19 |
| Location 9 | 0.12-0.46 | 0.27 | 0.17 | 2.59-4.82 | 3.96 | 1.20 | 36.3-49.1 | 42.03 | 6.5 | 4.80-6.82 | 5.52 | 1.13 | 1.25-8.50 | 5.92 | 4.05 | 3.50-4.00 | 3.75 | 0.35 |
| Location 10 | 0.08-0.45 | 0.25 | 0.19 | 2.54-4.96 | 4.11 | 1.36 | 36.3-62.3 | 48.27 | 13.1 | 4.20-6.30 | 5.39 | 1.08 | 1.25-6.66 | 4.25 | 2.75 | 3.00-11.50 | 7.25 | 6.01 |
| Location 11 | 0.05-0.52 | 0.26 | 0.24 | 2.50-5.50 | 3.92 | 1.51 | 44.3-79.6 | 59.27 | 18.3 | 4.00-8.50 | 6.64 | 2.35 | 1.50-4.00 | 3.02 | 1.33 | 2.25-2.59 | 2.38 | 0.18 |
| Location 12 | 0.14-0.73 | 0.30 | 0.38 | 4.08-5..47 | 4.92 | 0.74 | 76.4-77.4 | 80.33 | 6.0 | 2.75-10.40 | 6.98 | 3.89 | 2.05-17.00 | 8.02 | 7.92 | 2.00-42.00 | 22.00 | 28.2 |
| Location 13 | 0.04-0.43 | 0.20 | 0.21 | 2.98-3.81 | 3.50 | 0.46 | 29.88-66.3 | 45.26 | 18.9 | 5.20-8.18 | 6.49 | 1.53 | 1.50-6.33 | 3.59 | 2.48 | 1.50-15.50 | 8.50 | 9.90 |
| Location 14 | 0.03-0.69 | 0.29 | 0.35 | 3.54-5.06 | 4.38 | 0.77 | 32.98-68.9 | 51.56 | 18.0 | 6.40-9.25 | 7.85 | 1.43 | 8.90-14.33 | 10.74 | 3.11 | 4.25-5.00 | 4.63 | 0.53 |
| Location 15 | 0.07-0.68 | 0.28 | 0.35 | 3.55-5.16 | 3.94 | 1.08 | 41.52-74.3 | 57.34 | 16.4 | 9.60-19.15 | 13.45 | 5.04 | 1.60-33.00 | 13.70 | 16.8 | 4.50-10.00 | 7.25 | 3.89 |
| Location 16 | 0.81-1.01 | 0.89 | 0.10 | 3.23-4.16 | 3.70 | 0.47 | 37.82-79.6 | 58.94 | 20.9 | 6.50-17.80 | 11.95 | 5.66 | 1.45-41.33 | 16.01 | 22.0 | 12.00-43.25 | 27.63 | 22.1 |
| Location 17 | 0.04-0.43 | 0.20 | 0.21 | 2.39-2.78 | 2.59 | 0.20 | 29.98-53.6 | 39.96 | 12.2 | 5.22-9.60 | 7.32 | 2.20 | 0.50-27.66 | 9.97 | 15.3 | 1.00-20.75 | 10.88 | 13.9 |
| Location 18 | 0.01-0.13 | 0.08 | 0.08 | 2.67-7.25 | 4.96 | 3.24 | 45.2-68.6 | 56.90 | 16.6 | 4.75-8.40 | 6.58 | 2.58 | 5.15-10.00 | 7.58 | 3.43 | 1.5 | 1.50 | 0.00 |
| Location 19 | 0.01-0.22 | 0.12 | 0.15 | 4.46-6.21 | 5.34 | 1.24 | 36.3-62.3 | 49.30 | 18.4 | 5.91-10.40 | 8.16 | 3.17 | 1.66-3.10 | 2.38 | 1.02 | 1.75 | 1.75 | 0.00 |
| Location 20 | 0.13-0.59 | 0.30 | 0.26 | 5.94-9.68 | 7.62 | 1.90 | 34.4-71.8 | 47.87 | 20.8 | 4.90-9.20 | 6.73 | 2.22 | 1.75-2.95 | 2.23 | 0.63 | 3.25-6.00 | 4.63 | 1.94 |
| Location 21 | 0.05-0.44 | 0.27 | 0.20 | 5.92-13.76 | 9.41 | 3.99 | 26.8-59.6 | 40.90 | 16.9 | 4.10-10.00 | 6.78 | 2.99 | 2.00-4.05 | 2.90 | 1.05 | 1.50-2.75 | 2.13 | 0.88 |
| Location 22 | 0.01-0.02 | 0.02 | 0.01 | 2.40-6.08 | 4.24 | 2.60 | 61.6-88.4 | 75.00 | 19.0 | 5.93-9.40 | 7.67 | 2.45 | 2.33-3.55 | 2.94 | 0.86 | 4 | 4.00 | 0.00 |
| Location 23 | 0.09-0.11 | 0.10 | 0.02 | 3.77-7.79 | 5.78 | 2.84 | 103.-132.6 | 118.0 | 20.7 | 4.35-14.00 | 9.18 | 6.82 | 1.66-4.65 | 3.16 | 2.11 | 0.5 | 0.50 | 0.00 |
| Location 24 | 0.20-1.02 | 0.62 | 0.58 | 6.12-9.89 | 8.00 | 2.67 | 34.2-67.6 | 50.90 | 23.6 | 6.80-8.45 | 7.63 | 1.17 | 1.00-3.66 | 2.33 | 1.88 | 0.75-2.50 | 1.63 | 1.24 |
| Location 25 | 0.27-0.55 | 0.41 | 0.20 | 7.25-5.59 | 6.42 | 1.17 | 89.3-94.3 | 91.80 | 3.5 | 9.80-12.65 | 11.23 | 2.02 | 5.00-8.75 | 6.88 | 2.65 | 2.00-10.00 | 6.00 | 5.66 |

**Table S7.** Standardized qPCR statistics for MST and pathogen quantification assays.

| **Assay** | **Compiled**  **Slope** | **Compiled**  **Y-intercept** | **Compiled**  **R^2^ value** | **Compiled**  **Efficiency** | **LOQ**  **(cp/µl)** | **LOD**  **(cp/µl)** |
| --- | --- | --- | --- | --- | --- | --- |
| BacUni | -3.32 | 41.3 | 0.99 | 100.0 | 100^a^ | - |
| HF183 Taqman | -3.37 | 38.7 | 0.99 | 99.7 | 10 ^a^ | - |
| Pig-2-Bac | -3.27 | 41.1 | 0.99 | 102 | 30 ^a^ | - |
| GFD | -3.27 | 36.9 | 0.99 | 96 | 10 ^a^ | - |
| *Leptospira* (*LipL32*) | -3.33 | 40.06 | 0.99 | 99.38 | 10 ^b^ | 3 ^b^ |
| *Campylobacter* (*mapA*) | -3.22 | 39.3 | 0.99 | 104 | 10 ^b^ | 3 ^b^ |
| *Shigella* (*ipaH*) | -3.16 | 36.9 | 0.99 | 100.08 | 10 ^b^ | 3 ^b^ |
| *stx2* | -3.37 | 33.6 | 0.99 | 102. | 10 ^b^ | 3 ^b^ |
| *eae* | -3.34 | 34.7 | 0.99 | 98.9 | 10 ^b^ | 3 ^b^ |

LOD: Limit of Detection, LOQ: Limit of Quantification.

^a^ Based on MST validation study carried in this study.

^b^ Based on Oster et al. (2014).

**Data Processing:**

For MST assays, Data were classified as quantifiable if two or more replicates were above LOD.

For Pathogens, Data were classified as quantifiable (Q), detectable but not quantifiable (DNQ), and nondetectable (ND). A sample was classified as Q if two or more replicates were above the LOQ, DNQ if two or more replicates were between the LOD and LOQ, and ND if two or more replicates were below the LOD.

**Table S8.** Concentration of pathogenic bacterial marker genes in Tiaoxi river water and sediment samples.

| ***Campylobacter jejuni*** | | | | | | |
| --- | --- | --- | --- | --- | --- | --- |
|  | **Water (log_10_ gene copies/100ml)** | | | **Sediment (log_10_ gene copies/gram)** | | |
| **Location** | **Autumn 2014** | **Winter 2015** | **Summer 2015** | **Autumn 2014** | **Winter 2015** | **Summer 2015** |
| L-1 | DNQ | ND | **2.76** | ND | **3.75** | DNQ |
| L-2 | ND | ND | **2.66** | ND | DNQ | DNQ |
| L-3 | ND | DNQ | **2.77** | ND | ND | **3.31** |
| L-4 | ND | **2.49** | **2.52** | **3.37** | **3.71** | DNQ |
| L-5 | DNQ | ND | **2.63** | ND | **3.52** | **3.31** |
| L-6 | **2.32** | ND | **2.72** | DNQ | DNQ | DNQ |
| L-8 | ND | **2.38** | **2.60** | ND | **3.58** | **3.32** |
| L-10 | ND | DNQ | **2.76** | DNQ | ND | ND |
| L-12 | ND | **2.38** | **2.81** | ND | ND | DNQ |
| L-13 | ND | **2.31** | **2.72** | ND | **3.46** | DNQ |
| L-14 | ND | DNQ | **2.87** | DNQ | ND | **3.34** |
| L-15 | ND | ND | **2.79** | DNQ | **3.46** | ND |
| L-16 | DNQ | DNQ | **2.88** | ND | DNQ | ND |
| L-20 | ND | DNQ | **2.81** | **3.86** | ND | ND |
| L-21 | ND | ND | **2.75** | ND | **3.47** | DNQ |

DNQ: Detected not Quantifiable, ND: Not Detected.

| ***Leptospira* spp.** | | | | | | |
| --- | --- | --- | --- | --- | --- | --- |
|  | **Water (log_10_ gene copies/100ml)** | | | **Sediment (log_10_ gene copies/gram)** | | |
| **Location** | **Autumn 2014** | **Winter 2015** | **Summer 2015** | **Autumn 2014** | **Winter 2015** | **Summer 2015** |
| L-1 | **2.46** | **2.54** | ND | ND | ND | ND |
| L-2 | **2.59** | **2.67** | ND | ND | **3.33** | ND |
| L-3 | ND | ND | ND | ND | ND | ND |
| L-4 | ND | **2.52** | ND | ND | ND | ND |
| L-5 | ND | **2.61** | ND | ND | **3.52** | ND |
| L-6 | ND | **3.00** | ND | ND | ND | ND |
| L-8 | ND | ND | ND | ND | ND | ND |
| L-10 | **2.49** | **2.44** | ND | ND | ND | ND |
| L-12 | ND | ND | ND | ND | ND | ND |
| L-13 | ND | **2.44** | ND | ND | ND | ND |
| L-14 | ND | ND | ND | ND | **3.47** | ND |
| L-15 | ND | ND | ND | ND | **3.38** | ND |
| L-16 | ND | **2.54** | ND | ND | **3.45** | ND |
| L-20 | **2.55** | **2.82** | ND | ND | **3.65** | ND |
| L-21 | **2.44** | **3.13** | ND | ND | ND | ND |

| ***Shigella* spp.** | | | | | | |
| --- | --- | --- | --- | --- | --- | --- |
|  | **Water (log_10_ gene copies/100ml)** | | | **Sediment (log_10_ gene copies/gram)** | | |
| **Location** | **Autumn 2014** | **Winter 2015** | **Summer 2015** | **Autumn 2014** | **Winter 2015** | **Summer 2015** |
| L-1 | DNQ | ND | ND | **3.33** | DNQ | DNQ |
| L-2 | DNQ | ND | ND | **3.39** | DNQ | DNQ |
| L-3 | **2.33** | ND | ND | **3.34** | DNQ | DNQ |
| L-4 | DNQ | ND | ND | **3.40** | DNQ | DNQ |
| L-5 | ND | ND | ND | DNQ | DNQ | ND |
| L-6 | DNQ | ND | ND | **3.33** | DNQ | DNQ |
| L-8 | DNQ | ND | DNQ | **3.40** | ND | DNQ |
| L-10 | DNQ | DNQ | ND | **3.36** | DNQ | DNQ |
| L-12 | **2.35** | DNQ | ND | **3.48** | ND | DNQ |
| L-13 | DNQ | DNQ | ND | **3.41** | DNQ | DNQ |
| L-14 | DNQ | DNQ | ND | **3.42** | ND | DNQ |
| L-15 | DNQ | DNQ | DNQ | **3.43** | ND | DNQ |
| L-16 | DNQ | DNQ | ND | **3.44** | DNQ | DNQ |
| L-20 | DNQ | DNQ | ND | **3.43** | DNQ | DNQ |
| L-21 | DNQ | DNQ | DNQ | **3.40** | DNQ | DNQ |

| ***Shiga toxin-producing E.coli*** | | | | | | |
| --- | --- | --- | --- | --- | --- | --- |
|  | **Water (log_10_ gene copies/100ml)** | | | **Sediment (log_10_ gene copies/gram)** | | |
| **Location** | **Autumn 2014** | **Winter 2015** | **Summer 2015** | **Autumn 2014** | **Winter 2015** | **Summer 2015** |
| L-1 | ND | DNQ | ND | ND | DNQ | DNQ |
| L-2 | ND | DNQ | DNQ | DNQ | ND | ND |
| L-3 | ND | DNQ | DNQ | **3.46** | ND | ND |
| L-4 | ND | DNQ | DNQ | **5.35** | ND | DNQ |
| L-5 | ND | **2.31** | DNQ | **5.12** | ND | ND |
| L-6 | ND | ND | DNQ | **5.32** | DNQ | **3.36** |
| L-8 | ND | ND | DNQ | **4.90** | DNQ | DNQ |
| L-10 | ND | ND | DNQ | **5.02** | ND | ND |
| L-12 | ND | DNQ | DNQ | **4.97** | ND | ND |
| L-13 | ND | DNQ | ND | **4.76** | ND | ND |
| L-14 | ND | DNQ | ND | **5.90** | DNQ | DNQ |
| L-15 | ND | DNQ | DNQ | **5.65** | DNQ | **3.41** |
| L-16 | ND | DNQ | DNQ | ND | DNQ | DNQ |
| L-20 | ND | DNQ | DNQ | **5.43** | DNQ | **3.33** |
| L-21 | ND | **2.42** | DNQ | ND | DNQ | DNQ |

| ***Escherichia coli* O157: H7** | | | | | | |
| --- | --- | --- | --- | --- | --- | --- |
|  | **Water (log_10_ gene copies/100ml)** | | | **Sediment (log_10_ gene copies/gram)** | | |
| **Location** | **Autumn 2014** | **Winter 2015** | **Summer 2015** | **Autumn 2014** | **Winter 2015** | **Summer 2015** |
| L-1 | ND | ND | ND | ND | ND | ND |
| L-2 | ND | ND | ND | ND | ND | ND |
| L-3 | ND | ND | ND | ND | ND | ND |
| L-4 | ND | ND | ND | ND | ND | ND |
| L-5 | ND | ND | ND | ND | ND | ND |
| L-6 | ND | ND | ND | ND | ND | ND |
| L-8 | ND | ND | ND | ND | ND | ND |
| L-10 | ND | ND | ND | ND | ND | ND |
| L-12 | ND | ND | ND | DNQ | ND | ND |
| L-13 | ND | ND | ND | **3.32** | ND | ND |
| L-14 | ND | ND | ND | **3.45** | ND | ND |
| L-15 | ND | ND | ND | DNQ | ND | ND |
| L-16 | ND | ND | ND | ND | ND | ND |
| L-20 | ND | ND | ND | **4.03** | ND | ND |
| L-21 | ND | ND | ND | ND | ND | ND |

**Table S9.** GPS coordinates of some of the pig and poultry farms at location 13 and 21.

| Sampling Location | potential source/farm type | Distance from sampling location | Latitude | Longitude |
| --- | --- | --- | --- | --- |
| L-13 | Poultry farms | <500 m | N30°51′45.67″ | E120°04′10.66″ |
| L-13 | Pig farms | <500 m | N30°51′44.25″ | E120°04′42.14″ |
| L-21 | Poultry farms | <2 km | N30°55′39.67″ | E119°54′45.23″ |
| L-21 | Pig farms | <2 km | N30°55'21.0" | E119°54'51.8" |

**References:**

Green, H.C., Dick, L.K., Gilpin, B., Samadpour, M., and Field, K.G. (2012). Genetic markers for rapid PCR-based identification of gull, Canada goose, duck, and chicken fecal contamination in water. *Appl Environ Microbiol* 78**,** 503-510.

Green, H.C., Haugland, R.A., Varma, M., Millen, H.T., Borchardt, M.A., Field, K.G., Walters, W.A., Knight, R., Sivaganesan, M., Kelty, C.A., and Shanks, O.C. (2014). Improved HF183 Quantitative Real-Time PCR Assay for Characterization of Human Fecal Pollution in Ambient Surface Water Samples. *Applied and Environmental Microbiology* 80**,** 3086-3094.

Kildare, B.J., Leutenegger, C.M., Mcswain, B.S., Bambic, D.G., Rajal, V.B., and Wuertz, S. (2007). 16S rRNA-based assays for quantitative detection of universal, human-, cow-, and dog-specific fecal Bacteroidales: a Bayesian approach. *Water Res* 41**,** 3701-3715.

Mieszkin, S., Furet, J.P., Corthier, G., and Gourmelon, M. (2009). Estimation of pig fecal contamination in a river catchment by real-time PCR using two pig-specific Bacteroidales 16S rRNA genetic markers. *Appl Environ Microbiol* 75**,** 3045-3054.

Odagiri, M., Schriewer, A., Hanley, K., Wuertz, S., Misra, P.R., Panigrahi, P., and Jenkins, M.W. (2015). Validation of Bacteroidales quantitative PCR assays targeting human and animal fecal contamination in the public and domestic domains in India. *Sci Total Environ* 502**,** 462-470.

Ohad, S., Ben-Dor, S., Prilusky, J., Kravitz, V., Dassa, B., Chalifa-Caspi, T., Kashi, Y., and Rorman, E. (2016). The Development of a Novel qPCR Assay-Set for Identifying Fecal Contamination Originating from Domestic Fowls and Waterfowl in Israel. *Frontiers in Microbiology* 7.

Oster, R.J., Wijesinghe, R.U., Haack, S.K., Fogarty, L.R., Tucker, T.R., and Riley, S.C. (2014). Bacterial pathogen gene abundance and relation to recreational water quality at seven Great Lakes beaches. *Environ Sci Technol* 48**,** 14148-14157.

Reischer, G.H., Ebdon, J.E., Bauer, J.M., Schuster, N., Ahmed, W., Astrom, J., Blanch, A.R., Bloschl, G., Byamukama, D., Coakley, T., Ferguson, C., Goshu, G., Ko, G., De Roda Husman, A.M., Mushi, D., Poma, R., Pradhan, B., Rajal, V., Schade, M.A., Sommer, R., Taylor, H., Toth, E.M., Vrajmasu, V., Wuertz, S., Mach, R.L., and Farnleitner, A.H. (2013). Performance characteristics of qPCR assays targeting human- and ruminant-associated bacteroidetes for microbial source tracking across sixteen countries on six continents. *Environ Sci Technol* 47**,** 8548-8556.

Seurinck, S., Defoirdt, T., Verstraete, W., and Siciliano, S.D. (2005). Detection and quantification of the human-specific HF183 Bacteroides 16S rRNA genetic marker with real-time PCR for assessment of human faecal pollution in freshwater. *Environ Microbiol* 7**,** 249-259.

Shanks, O.C., Kelty, C.A., Sivaganesan, M., Varma, M., and Haugland, R.A. (2009). Quantitative PCR for genetic markers of human fecal pollution. *Appl Environ Microbiol* 75**,** 5507-5513.

Siefring, S., Varma, M., Atikovic, E., Wymer, L., and Haugland, R.A. (2008). Improved real-time PCR assays for the detection of fecal indicator bacteria in surface waters with different instrument and reagent systems. *J Water Health* 6**,** 225-237.

Vadde, K., Wang, J., Cao, L., Yuan, T., Mccarthy, A., and Sekar, R. (2018). Assessment of Water Quality and Identification of Pollution Risk Locations in Tiaoxi River (Taihu Watershed), China. *Water* 10**,** 183.

.
